# A chemical probe inhibitor targeting STAT1 restricts cancer stem cell traits and angiogenesis in colorectal cancer

**DOI:** 10.1186/s12929-022-00803-4

**Published:** 2022-03-22

**Authors:** Pei-Hsuan Chou, Cong-Kai Luo, Niaz Wali, Wen-Yen Lin, Shang-Kok Ng, Chun-Hao Wang, Mingtao Zhao, Sheng-Wei Lin, Pei-Ming Yang, Pin-Jung Liu, Jiun-Jie Shie, Tzu-Tang Wei

**Affiliations:** 1 Department and Graduate Institute of Pharmacology, College of Medicine, National Taiwan University, No. 1, Jen-Ai Road, 1st Section, Taipei, 10051 Taiwan; 2 Institute of Chemistry, Academia Sinica, 128 Academia Road, Section 2, Taipei, 11529 Taiwan; 3 Institute of Biochemical Sciences, College of Life Science, National Taiwan University, Taipei, 10617 Taiwan; 4 Chemical Biology and Molecular Biophysics, Taiwan International Graduate Program in Chemical Biology and Molecular Biophysics (TIGP-CBMB), Academia Sinica, Taipei, 11529 Taiwan; 5 School of Pharmacy, College of Medicine, National Taiwan University, Taipei, 10051 Taiwan; 6 School of Medicine, College of Medicine, National Taiwan University, Taipei, 10051 Taiwan; 7 Center for Cardiovascular Research, The Abigail Wexner Research Institute, Nationwide Children’s Hospital, Columbus, OH 43210 USA; 8 The Heart Center, Nationwide Children’s Hospital, Columbus, OH 43210 USA; 9 Department of Pediatrics, College of Medicine, The Ohio State University, Columbus, OH 43210 USA; 10 Institute of Biological Chemistry, Academia Sinica, Taipei, 11529 Taiwan; 11 Graduate Institute of Cancer Biology and Drug Discovery, College of Medical Science and Technology, Taipei Medical University, Taipei, 11031 Taiwan; 12 School of Pharmacy, College of Pharmacy, Taipei Medical University, Taipei, 11031 Taiwan

**Keywords:** STAT1, Colorectal cancer, Stemness, Angiogenesis, 4’,5,7-trihydroxyisoflavone (THIF), BODIPY-THIF

## Abstract

**Background:**

Colorectal cancer (CRC) is a worldwide cancer with rising annual incidence. New medications for patients with CRC are still needed. Recently, fluorescent chemical probes have been developed for cancer imaging and therapy. Signal transducer and activator of transcription 1 (STAT1) has complex functions in tumorigenesis and its role in CRC still needs further investigation.

**Methods:**

RNA sequencing datasets in the NCBI GEO repository were analyzed to investigate the expression of STAT1 in patients with CRC. Xenograft mouse models, tail vein injection mouse models, and azoxymethane/dextran sodium sulfate (AOM/DSS) mouse models were generated to study the roles of STAT1 in CRC. A ligand-based high-throughput virtual screening approach combined with SWEETLEAD chemical database analysis was used to discover new STAT1 inhibitors. A newly designed and synthesized fluorescently labeled 4’,5,7-trihydroxyisoflavone (THIF) probe (BODIPY-THIF) elucidated the mechanistic actions of STAT1 and THIF in vitro and in vivo. Colonosphere formation assay and chick chorioallantoic membrane assay were used to evaluate stemness and angiogenesis, respectively.

**Results:**

Upregulation of STAT1 was observed in patients with CRC and in mouse models of AOM/DSS-induced CRC and metastatic CRC. Knockout of STAT1 in CRC cells reduced tumor growth in vivo. We then combined a high-throughput virtual screening approach and analysis of the SWEETLEAD chemical database and found that THIF, a flavonoid abundant in soybeans, was a novel STAT1 inhibitor. THIF inhibited STAT1 phosphorylation and might bind to the STAT1 SH2 domain, leading to blockade of STAT1-STAT1 dimerization. The results of in vitro and in vivo binding studies of THIF and STAT1 were validated. The pharmacological treatment with BODIPY-THIF or ablation of STAT1 via a CRISPR/Cas9-based strategy abolished stemness and angiogenesis in CRC. Oral administration of BODIPY-THIF attenuated colitis symptoms and tumor growth in the mouse model of AOM/DSS-induced CRC.

**Conclusions:**

This study demonstrates that STAT1 plays an oncogenic role in CRC. BODIPY-THIF is a new chemical probe inhibitor of STAT1 that reduces stemness and angiogenesis in CRC. BODIPY-THIF can be a potential tool for CRC therapy as well as cancer cell imaging.

**Supplementary Information:**

The online version contains supplementary material available at 10.1186/s12929-022-00803-4.

## Background

Colorectal cancer (CRC) occurs worldwide and has an increasing annual incidence [1]. Metastasis, which occurs most frequently in the liver and lung, is a leading cause of death from CRC [2]. Metastatic CRC is an incurable disease requiring systemic drug therapy to prolong the survival of patients. Although the gradual discovery of chemotherapeutic and molecular targeted agents has improved the survival of patients with metastatic CRC from 12 to 24 months in the past two decades, inevitably, the emergence of acquired resistance ultimately leads to mortality [3–5]. This limitation introduces an unmet medical need for the discovery of new therapeutics for CRC.

The signal transducer and activator of transcription (STAT) protein is an essential component of interferon (IFN)/Janus kinase (JAK) signaling [6]. STAT proteins contain an N-terminal domain, a coiled-coil domain, a DNA-binding domain, an alpha-helical linker domain, an SH2 domain, and a transactivation domain [7]. The SH2 domain is necessary for receptor association and tyrosine phosphodimer formation. There are seven members of the STAT family, namely, STAT1, STAT2, STAT3, STAT4, STAT5A, STAT5B, and STAT6, and all are involved in homeostasis, immune surveillance, and defense [8]. Among STAT family members, STAT3 is the most well-studied member; it is overexpressed in the majority of human cancers, and targeting STAT3 signaling has been recognized as a potential strategy for treating cancer owing to its roles in tumor formation, metastasis, and drug resistance [9, 10]. In addition to STAT3, STAT1 is the first discovered member of the STAT family, but its role in tumorigenesis is less understood. In the canonical signaling pathway, STAT1 is initially phosphorylated and activated by receptor-activated kinases such as JAK in response to ligand stimulation [11]. Phosphorylated STAT1 forms homodimers or heterodimers with other STAT family members, such as STAT3, and then translocates from the cytosol to the nucleus, where it acts as a transcription factor [12]. STAT1 is generally considered a tumor suppressor because it plays an essential role in the immune response and protects against pathogen infections [13, 14]. However, aberrant STAT1 activation has been found in several types of human cancer [15–18]. There is also increasing evidence supporting the tumor-promoting functions of STAT1 [19]. These reports indicate that STAT1 may perform complex functions in tumorigenesis, and its role in different cancer types still needs further investigation.

One of the steps in the early stage of new drug discovery is identifying active ingredients as lead compounds that can be further optimized into drug candidates [20]. The combination of virtual screening approaches with chemical database analysis has been widely used in the pharmaceutical industry for the discovery of new drugs [21]. Ligand-based and structure-based methods are the main approaches for computer-aided virtual screening strategies [22]. Structure-based virtual screening is a useful approach that employs information on the crystal structure of the target of interest in the design of new lead compounds via large-scale molecular docking experiments [23]. This approach identifies the docking poses and binding affinities of the input compounds to the target of interest. Ligand-based virtual screening is based on shape similarity and volume similarity between two compounds [24]. Models merging ligand-based and structure-based approaches are considered more effective than independent models for new drug discovery [25].

In this study, we performed a combined strategy of ligand-based and structure-based virtual screening to identify new STAT1 inhibitors. ROCS software was used for high-throughput virtual screening of the SWEETLEAD chemoinformatics database as the chemical library. We discovered that 4’,5,7-trihydroxyisoflavone (THIF), an isoflavone abundant in soybeans, can bind to the STAT1 protein and block the STAT1-STAT1 interaction. Complementary in vitro and in vivo studies revealed that THIF is a new STAT1 inhibitor that directly binds to the STAT1 SH2 domain. Chemical probes with a high fluorescence signal are of use in cancer imaging development and play important roles in the study of drug distribution and metabolism in cancers [26, 27]. To investigate the in vitro and in vivo biodistribution of STAT1 in CRC, we then designed and synthesized a fluorescently labeled THIF probe (BODIPY-THIF). An IVIS imaging system was used to study the biodistribution of BODIPY-THIF in AOM/DSS mouse models.

## Methods

### Reagents

Delta-9-tetrahydrocannabinol (Δ^9^-THC) was obtained from Cerilliant (Round Rock, TX, USA). Fludarabine was obtained from Selleck Chemicals (Houston, TX, USA). 3-(4,5-dimethylthiazol-2-yl)-2,5-diphenyl tetrazolium bromide (MTT) and dimethyl sulfoxide (DMSO) were purchased from Sigma-Aldrich (Burlington, MA, USA). Azoxymethane (AOM) was purchased from Sigma. Dextran sulfate sodium (DSS) was purchased from MP Biomedicals (Solon, OH, USA). NE-PER Nuclear and Cytoplasmic Extraction Reagents were purchased from Thermo Fisher Scientific (Waltham, MA, USA). Recombinant human IFN-γ was purchased from PeproTech (Rocky Hill, NJ, USA). Recombinant human STAT1 protein was purchased from ABclonal Technology (Woburn, MA, USA). Directed in vivo angiogenesis assay (DIVAA) Kit was purchased from Trevigen (Minneapolis, MN, USA).

### Patients and tumor specimens

Tissues from 12 CRC patients used in our previous study were obtained [28]. The control tissues were from the normal mucosa adjacent to tumors. All samples were stored at -80 °C. The Institutional Review Board of National Taiwan University Hospital approved the procedures for tissue collection and analysis, and written informed consent was obtained from each patient.

### Immunohistochemistry (IHC) assay

IHC was performed with a One-step Polymer-HRP Detection Kit (Biogenex, Fremont, CA, USA) on sections from 10% paraffin-embedded samples according to the manufacturer’s protocols. Images were acquired using a Tissue FAXS system (TissueGnostics, Los Angeles, CA, USA), and the results of DAB-positive cells were viewed.

### Cell culture

HCT116 human colon cancer cells were cultured in RPMI 1640 medium. All medium were supplemented with 10% heat-inactivated fetal bovine serum (GIBCO, 10437, Waltham, MA, USA), 1% l-glutamine (GIBCO, 25030), and 1% antibiotic:antimycotic solution (Gemini Bio-Products, 400–101, West Sacramento, CA, USA). Cells were incubated at 37 °C in a humidified incubator containing 20% O_2_ and 5% CO_2_.

### Cell viability assay

Cells were seeded at 3000 cells/well in 96-well plates, maintained for 14–16 h, and then treated with test compounds. After 48 h of incubation, cells were washed with PBS then added medium containing MTT reagent at a final concentration of 0.5 mg/mL for 4 h. Then, the medium was replaced with 200 μL of DMSO for 30 min. The absorbance at 570 nm was measured on a multiwell plate reader. The 50% of inhibition concentration (IC_50_) of death cells was calculated.

### Animal model of AOM/DSS-induced CRC

Seven-week-old male C57BL/6 mice were obtained from the National Laboratory Animal Center (Taiwan). CRC was induced by intraperitoneal injection of AOM (12.5 mg/kg) in conjunction with DSS stimulation. Mice were maintained on a regular diet and drinking water for 7 days and were then subjected to 3 cycles of DSS treatment, with each cycle consisting of the administration of 3.5% DSS for 5 days followed by a 14-day recovery period with regular water. Colon tissues were collected and fixed with formalin.

### Establishment of tail vein injection lung cancer models

Seven-week-old male NOD/SCID mice were obtained from the National Laboratory Animal Center. HCT116 cells (2.5 × 10^6^) were injected intravenously into the tail veins of the mice. After three weeks, the mice were sacrificed. Lung tissues were collected, weighed and fixed with formalin.

### Gene knockout (KO) using the CRISPR/Cas9 system

According to the manufacturer’s protocol, CRISPR plasmids (Santa Cruz Biotechnology, Dallas, TX, USA) were transfected with UltraCruz Transfection Reagent (Santa Cruz). Briefly, CRISPR plasmids expressing Cas9 and STAT1 sgRNAs were transfected into HCT116 cells for 12 h in 6-well plates. Successful transfection of the CRISPR/Cas9 KO plasmid was visually confirmed by the detection of green fluorescent protein (GFP). Cells were selected with puromycin (0.5 μg/mL) for 14 days. Single clones were picked from 96-well plates according to GFP expression and expanded to generate monoclonal cell colonies. The mRNA expression level of STAT1 was measured by qPCR and normalized to that of GAPDH.

### Animal model for CRC xenografts

Seven-week-old male BALB/c nude mice were subcutaneously injected with 1 × 10^7^ STAT1 wild-type (WT) HCT116 cells and STAT1 KO HCT116 cells (suspended in 100 μL of PBS) in the right rear flank and left rear flank, respectively. After three weeks, the mice were sacrificed. Tumor tissues were collected, weighed and fixed with formalin.

### High-throughput virtual screening

The molecular structures of 3 published STAT1 inhibitors (fludarabine, ISS840, and pravastatin) were used as lead compounds for high-throughput virtual screening. STAT1 inhibitors were desalted, and query structures were generated by BROOD software (version 3.0.0.3, OpenEye Scientific, Santa Fe, NM, USA). ROCS software (version 3.2.2.2, OpenEye Scientific) was used for high-throughput virtual screening of the SWEETLEAD chemoinformatics database as the chemical library (https://simtk.org/projects/sweetlead). The top hits were ranked by the ROCS shape and color similarity scores and were selected from the hits for each STAT1 inhibitor.

### Molecular docking analysis

Protein–ligand docking studies were performed with the crystal structure of human STAT1 (PDB: 1YVL) by Mcule 1-click docking software (Mcule, Palo Alto, CA, USA), and the docking scores of the test compounds were estimated. The Glide module was used to dock the flexible ligands to the rigid binding site without any constraints using the default settings. The resulting GlideScores were used to estimate and rank the binding energy of the ligands. Binding interactions between the test compounds and amino acid residues in the STAT1 crystal structure were visualized with Discovery Studio software (version 2020, BIOVIA, San Diego, CA, USA).

### Surface plasmon resonance (SPR) analysis

The binding ability of THIF to STAT1 protein was determined using a Biacore T200 surface plasmon resonance instrument, respectively. STAT1 was applied with sodium acetate buffer (pH 4.5) and immobilized on a CM5 sensor chip with an amine coupling kit (Cytiva, Marlborough, MA, USA). Then, two-fold serially diluted THIF, starting at a concentration of 10 µM was injected into the flow channels in running buffer (PBS, pH 7.4, 0.1% DMSO). Each condition was implemented at a flow rate of 30 µL/min for 120 s at 25 °C. The obtained signals were subtracted from the reference channel that had not been coated with STAT1. The results were plotted in a resonance unit against a time sensorgram and analyzed using the BIAevaluation software (GE Healthcare, Chicago, IL, USA).

### Real-time polymerase chain reaction (qPCR)

Total RNA was isolated using TRIzol reagent. Reverse transcription was performed with 2 µg of total RNA, which was reverse transcribed into cDNA using oligo-dT. Real time PCR was performed with cDNA samples using the ABI Prism 7900 Sequence Detection System (Applied Biosystems, San Francisco, CA, USA). The data were normalized by GAPDH.

### Preparation of nuclear and cytosolic extracts

HCT116 cells (1 × 10^6^) in 10-cm dishes were treated with 50 μM THIF for 6 h. After treatment, cells were washed with cold PBS, collected with a cell scraper, harvested by centrifugation, and then treated with an NE-PER nuclear and cytoplasmic extraction reagent kit to extract cytosolic and nuclear proteins.

### Immunoprecipitation (IP) assay

The IP assay was performed using the IP kit (BioVision, Milpitas, CA, USA). Briefly, total cell lysates (500 μg) were immunoprecipitated with anti-STAT1 antibody overnight at 4 °C. Then, 20 μL protein A/G agarose was added to each sample and incubated for 2 h at 4 °C. The immunoprecipitates were washed with PBS and separated using 10% SDS-PAGE. Next, Western blot analysis was performed using specific antibodies.

### Western blot analysis

Cells were lysed on ice. Total cell lysates were centrifuged at 13,000 rpm for 15 min at 4 °C and then subjected to SDS PAGE using adequate percentage polyacrylamide gels. Immunoblotting was performed using specific antibodies to evaluate the expression of different proteins (Additional file 1: Table S1).

### STAT1 reporter assay

293 T cells (2 × 10^5^ cells per well of 6-well plate) were transfected with STAT1-5'UTR Luciferase (pFL-SV40-STAT1-5’UTR was a gift from Dr. Ming-Chih Lai at Chang Guang University) (50 ng) using Lipofectamine 3000 (Life Technologies, Carlsbad, CA, USA). Firefly luciferase activity was measured 24 h after transfection. Cells were treated with ampicillin (100 ng/mL) for 14 days. STAT1 reporter cells were seeded at 3000 cells/well in 96-well plates and maintained for 14–16 h, then treated with test compounds for 24 h. The relative luminescence units (RLU) were recorded using a plate reader. The IC_50_ of STAT1 activity was calculated by SigmaPlot software (Systat Software).

### Plasmids

STAT1a Flag pRc/CMV (1–750, plasmid 8691) plasmids were purchased from Addgene (Cambridge, MA, USA). To generate STAT1-Flag (1–488) and STAT1-Flag (489–750) plasmids, STAT1a-Flag pRc/CMV (1–750) was amplified by PCR with the following primers: STAT1-Flag (1–488), 5’-ATAAGAATGCGGCCGCATGTCTCAGTGGTAC GAACTT-3’ and 5’-TGTGGGCCCTACTTGTCATCGTCGTCCTTGTAGTCCAGG AAGAAGGACAGAT-3’; STAT1-Flag (489–750), 5’-ACTATAGGGAGACCCAAGC TTATGACTCCACCATGTGCACGATG-3’ and 5’-TATAGAATAGGGCCCTCTAGA CTATACTGTGTTCATCATACTGTCGAATTC-3’. The PCR products were digested by NotI and ApaI and cloned into the NotI/ApaI-digested STAT1a Flag pRc/CMV (1–750) vector.

### Colonosphere assay and analyses of stemness markers

HCT116 cells (2 × 10^4^ cells/mL) were cultured in serum-free DMEM/F12 medium supplemented with 1% antibiotic:antimycotic Solution, 1% insulin-transferrin-selenium, 20 ng/mL EGF and 25 ng/mL bFGF. The formation of colonospheres was captured every two days for 10 days.

### Chromatin IP and quantitative PCR (ChIP-qPCR) assay

HCT116 cells were treated with 5 μM Δ^9^-THC or 5 μM Δ^9^-THC plus 50 μM BODIPY-THIF for 6 h, cross-linked with 2% formaldehyde for 15 min, collected by scraping in PBS and then centrifuged and lysed in 1 mL of IP buffer (150 mM NaCl, 50 mM Tris–HCl pH 7.5, 5 mM EDTA, 0.5% Nonidet P-40 and 1% Triton X-100) containing protease inhibitors. The obtained nuclear pellet was resuspended in IP buffer and sonicated. ChIP assays using control rabbit IgG (Santa Cruz) or anti-STAT1 antibodies (Cell Signaling Technology, Danvers, MA, USA) were performed. The immunoprecipitated DNA and input DNA were extracted by incubating the samples with 100 μL of 10% Chelex (Bio-Rad Laboratories, Hercules, CA, USA), then boiling them to reverse the cross-linking and then centrifuging them to remove the Chelex slurry. PCR was performed using two oligonucleotide primers as follows: GDNF (forward primer, 5’-CAGCATGGAAATGAAGCCTA-3’; reverse primer, 5’- TAGTTTAGTCCCCAGGCTAG-3’); IGFBP6 (forward primer, 5’- TGCTGACAATGAGGTTCGTAT-3’; reverse primer, 5’- GTTATGCAACAGGGACCATC-3’); IGF2 (forward primer, 5’- CTGAATTCTCTAGAACGGGCATTCAGCA-3’; reverse primer, 5’- GGGGGCAGGGAGCCGCAGAG-3’); SCF (forward primer, 5’-ATAGGCTAGCAGCACAGACTTCCCTCCACAAAGT-3’; reverse primer, 5’-CATGGAAGCTTTGTGGCGACTCCGTTTAGCT-3’); and VEGFA (forward primer, 5’-GCGTGTCTCTGGACAGAGTTT-3’; reverse primer, 5’- AGCCTCAGCCCTTCCACA-3’).

### Chick chorioallantoic membrane (CAM) angiogenesis assay

All procedures involving chicken embryos were performed in agreement with the Institutional Animal Care and Use Committee of the College of Medicine, National Taiwan University. HCT116 cells were treated with vehicle (control) or BODIPY-THIF (50 μM or 100 μM) for 48 h, and the CM was collected. Fertilized chicken eggs were incubated at 37 °C in a humidified egg incubator. On day 8 of incubation, CAM was added to sterilized paper disks (5.5 mm). The disks were saturated with the CM of HCT116 cells. After 48 h, CAM vessels were photographed with a stereomicroscope connected to a camera. Angiogenesis was quantified as the number of vessel branch points contained in a circular region described by the filter disk. Images were quantitatively analyzed with WimCAM image analysis software (Wimasis, Calle la Palma, Cordoba, Spain).

### Directed in vivo angiogenesis assay

The angiogenesis ability was determined using a DIVAA kit (Trevigen). Briefly, small cylindrical silicon angioreactors filled with basement membrane extract containing the conditioned medium (CM) of HCT116 cells were implanted subcutaneously into both flanks of nude mice (two reactors per side, four reactors per animal). After 14 days, the reactors were excised, and the contents were processed as recommended by the manufacturer. Quantification of directed in vivo angiogenesis was assessed by fluorescein isothiocyanate dextran.

### Statistical analysis

SPSS (SPSS Inc.) was used for statistical analyses, which included Student’s *t*-tests, with statistical significance defined as *p* < 0.05 (* or ^#^), *p* < 0.01 (** or ^##^) and *p* < 0.001 (*** or ^###^). The data shown are representative of at least three independent experiments. Quantitative data are presented as the mean ± standard deviation (SD) values.

## Results

### STAT1 is upregulated in colorectal cancer

**Fig. 1:**
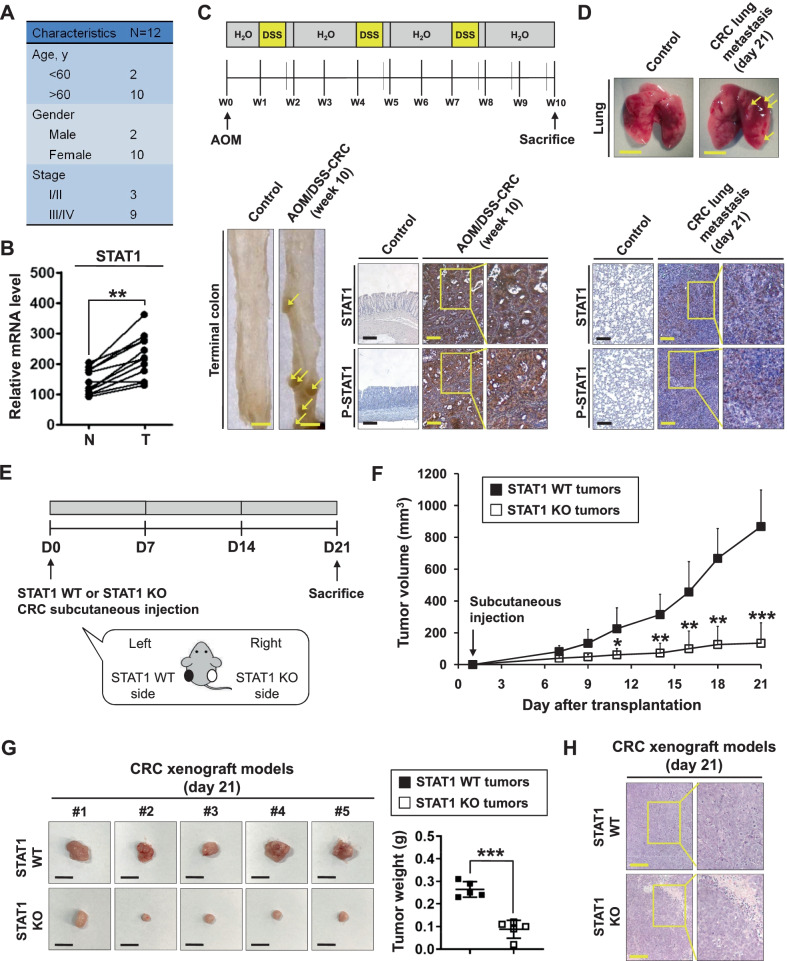
Expression levels of STAT1 in colorectal cancer. **A** Characteristics of 12 patients with CRC. **B** The mRNA expression level of STAT1 in CRC tissues from patients (T) normalized to that in the adjacent normal epithelium (N) is shown. Colon tissues from patients with CRC were collected, and gene expression was measured by qPCR and normalized to GAPDH expression. **p < 0.01. **C** Schematic overview of the experimental design (upper panel). Colitis-associated CRC was induced in B6 mice by AOM/DSS treatment (N = 8). The untreated control mice (N = 5) were littermates of similar age. Representative whole colons are shown, and the arrowhead indicates macroscopic polyps (lower left panel). Scale bars: 5 mm. Colon sections from healthy and AOM/DSS-treated mice were immunostained with specific antibodies (lower right panel). Scale bar: 250 μm. **D** Male NOD/SCID mice were intravenously injected with HCT116 cells (1 × 10^6^) through the tail vein. Mice were sacrificed at 3 weeks, and lung segments were fixed with formalin. Gross images of the lungs are shown. The arrowhead indicates the macroscopic lesions (upper panel). Scale bar: 5 mm. Lung sections from healthy and tail vein-injected mice were immunostained with specific antibodies (lower panel). Scale bar: 250 μm. **E**–**H** Role of STAT1 in colorectal tumor growth. STAT1 WT and STAT1 KO HCT116 cells (1 × 10^7^ cells) were subcutaneously implanted into male nude mice. The tumors were measured every two days for three weeks. **E** Schematic overview of the experimental design for the mouse models. **F** The tumor volume was calculated as follows: V = 0.5 × (length of the longest diameter) × (length of the shortest diameter)^2^. *p < 0.05; **p < 0.01; ***p < 0.001. **G** Gross images of xenograft tumors are shown (left panel). Scale bar: 1 cm. At the end of the experiment, the excised tumors were weighed (right panel). ***p < 0.001. **H** Tumor sections from the mouse models were counterstained with H&E, and high-magnification images of the areas in the yellow boxes are shown. Scale bars: 250 μm

To investigate the roles of STAT1 in CRC, we extracted gene expression microarray datasets from the Gene Expression Omnibus (GEO) (https://www.ncbi.nlm.nih.gov/gds/). Two gene expression profiles were used: GSE32323 and GSE10961 (Additional file 1: Fig. S1A). In the GSE32323 datasets, RNA sample data from paired colorectal tumors and control tissues of 17 CRC patients were extracted, and the expression of STAT1 was found to be higher in adenomas than in normal tissues (Additional file 1: Fig. S1B). In the GSE10961 datasets, RNA samples were extracted from liver metastases of 18 CRC patients and showed that the expression of STAT1 was higher in metastatic tissues than in primary tumors (Additional file 1: Fig. S1C). The expression of STAT1 was increased in human colon adenocarcinoma cells compared to normal human colon epithelial cells (Additional file 1: Fig. S1D). Paired tumor and control tissue samples were also collected from CRC patients at National Taiwan University Hospital (Fig. 1A). The mRNA expression of STAT1 was increased in CRC tissues compared to the adjacent normal epithelium (Fig. 1B).

Colon adenocarcinoma with dysplasia was observed in the mouse model of AOM/DSS-induced CRC (Additional file 1: Fig. S1E), and the expression of STAT1 in colorectal tumor tissues was evaluated by IHC staining. The expression of STAT1 and phosphorylated-STAT1 was increased in AOM/DSS-induced CRC tissues (Fig. 1C). To examine the expression of STAT1 in metastatic CRC tissues, HCT116 human CRC cells were intravenously injected into nude mice via the tail vein. Lung tumor nodules were found by macroscopic observation, and the expression of STAT1 and phosphorylated-STAT1 was found to be upregulated in metastatic tumors in the lungs (Additional file 1: Fig. S1F; Fig. 1D). These results suggest that STAT1 is highly expressed in both primary and metastatic CRC tissues and that it may play roles in the progression of CRC.

### STAT1 KO reduces colorectal tumor growth in vivo

Since STAT1 is upregulated in both primary and metastatic CRC tumor tissues, the role of STAT1 in CRC tumor growth was studied. The CRISPR/Cas9 system was utilized to generate stable STAT1 KO clones in human CRC cells (Additional file 1: Fig. S2A). STAT1 KO HCT116 cells could be identified by the GFP signal, and qPCR analysis confirmed the KO efficiency (Additional file 1: Fig. S2B-D). STAT1 KO also reduced the growth of CRC cells (Additional file 1: Fig. S2E).

To further study the in vivo role of STAT1 in CRC, a xenograft mouse model was established (Fig. 1E). STAT1 KO did not influence body weight (Additional file 1: Fig. S2F). We found that STAT1 KO reduced HCT116 tumor growth (Fig. 1F, G). H&E staining confirmed colorectal carcinoma in vivo (Fig. 1H). These results demonstrate the oncogenic role of STAT1 in CRC.

### High-throughput virtual screening of the SWEETLEAD database reveals that THIF exhibits anti-STAT1 activity

**Fig. 2:**
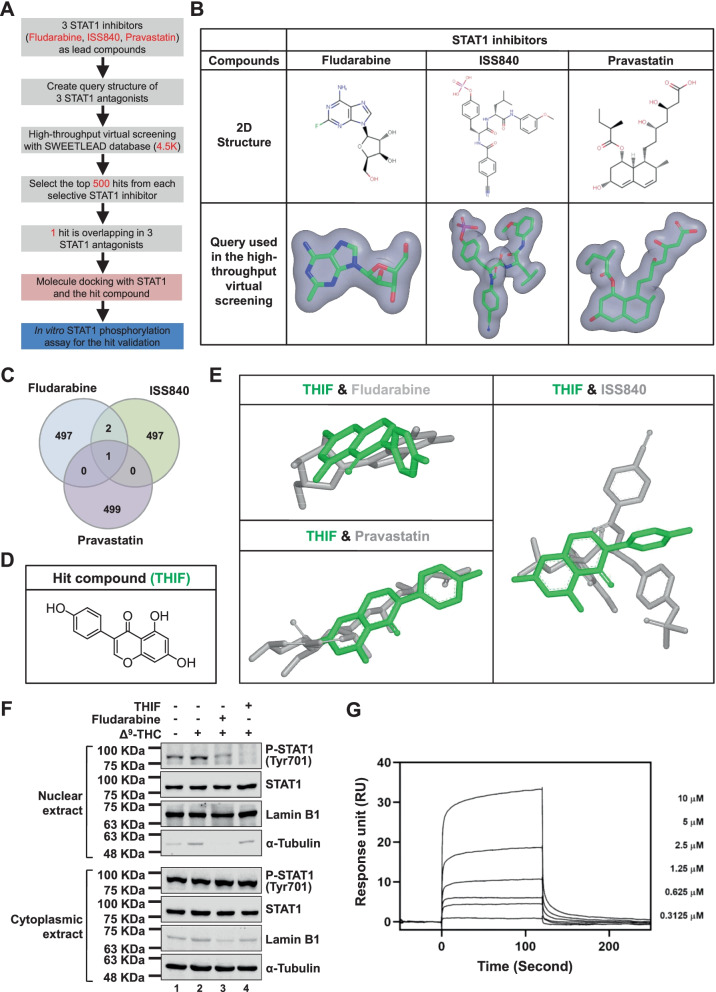
Identification of STAT1 inhibitors by high-throughput virtual screening. **A** High-throughput virtual screening workflow for discovering new STAT1 inhibitors. **B** The query structure of 3 published STAT1 inhibitors (fludarabine, ISS840 and pravastatin) was used for ligand-based high-throughput virtual screening. **C** Venn diagram showing the overlap between the 500 highest-scoring hits resulting from virtual screening of 3 published STAT1 inhibitors. **D** Molecular structure of hit compound THIF. **E** THIF shared structural homology with 3 published STAT1 inhibitors in ligand-based virtual screening. **F** Effect of THIF on STAT1 phosphorylation in CRC cells. HCT116 cells were treated with 5 μM Δ^9^-THC, Δ^9^-THC plus 10 μM THIF or Δ^9^-THC plus 10 μM fludarabine for 12 h. Protein extracts from the nuclear and cytoplasmic fractions were prepared. Western blot analysis was performed using the indicated antibodies. Lamin B1 served as the nuclear marker, and α-tubulin served as the cytosolic marker. **G** Direct interaction between STAT1 and THIF by SPR in vitro assay. Sensorgram of SPR for evaluating the binding affinity between STAT1 and THIF (equilibrium dissociation rate constant; *K*_D_ = 3.4 × 10^–5^ M)

ROCS OpenEye is a virtual screening software used to identify potentially active compounds through ligand shape comparison (https://www.eyesopen.com/). ROCS software has been widely employed for drug discovery [29]. Since STAT1 may play an oncogenic role in CRC, a ligand-based high-throughput virtual screening approach combined with chemical database analysis was used to discover new STAT1 inhibitors (Fig. 2A). The SWEETLEAD chemical database was screened against 3 published STAT1 inhibitors using the ROCS software suite (Fig. 2B). We found that THIF, a flavonoid abundant in soybeans, exhibited structural homology with the published STAT1 inhibitors (Fig. 2C, D). THIF adopted a shape similar to those of the STAT1 inhibitor query structures (Fig. 2E).

Specifically, STAT1 can be activated by several ligands, such as IFN, epidermal growth factor, platelet-derived growth factor, interleukin-6, and interleukin-27 [30]. Our previous study showed that Δ^9^-THC, the main psychoactive component in marijuana, is a specific STAT1 activator in CRC [31]. Western blot analysis revealed that Δ^9^-THC or IFN-γ-induced phosphorylation of STAT1 in CRC was inhibited by THIF (Fig. 2F; Additional file 1: Fig. S3A, B). Interestingly, THIF did not affect the protein expression level, mRNA level or reporter activity of STAT1 (Additional file 1: Fig. S3C-E). JAK2 and SHP2 are a STAT1 kinase and phosphatase, respectively [32], and we found that THIF did not influence the phosphorylation of either JAK2 or SHP2 (Additional file 1: Fig. S3F).

### THIF can bind to the STAT1 SH2 domain and block STAT1-STAT1 dimerization

**Fig. 3:**
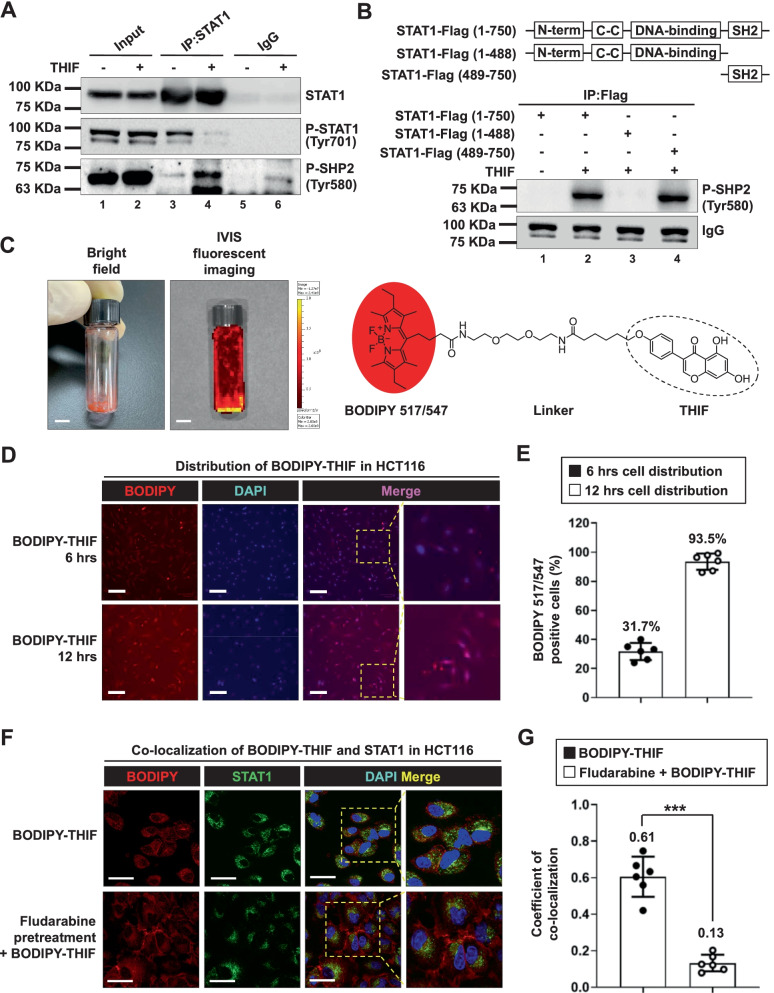
In vitro and in vivo binding of THIF to STAT1. **A** THIF blocks the STAT1-STAT1 interaction in CRC cells. HCT116 cells were treated with 50 μM THIF or vehicle (control) for 6 h, and proteins were isolated and immunoprecipitated with the indicated antibodies. **B** Schematic diagram of the STAT1 constructs (upper panel). The indicated plasmids (3 μg) were transfected into 293 T cells. Twenty-four hours later, the cells were treated with 50 μM THIF for 6 h. After incubation, cells were collected and used for IP experiments. Western blot analysis was performed using the indicated antibodies (lower panel). **C** Bright-field images and fluorescence images of BODIPY-THIF (5 mM) were acquired with an IVIS imaging system (left panel). Chemical structure of fluorescently labeled THIF (BODIPY-THIF) (right panel). The core structures of the BODIPY fluorophore are highlighted in red. **D** The cellular distribution of BODIPY-THIF in CRC cells. HCT116 cells were treated with BODIPY-THIF (50 μM) for 6 or 12 h and were then subjected to fluorescence microscopy to detect BODIPY-THIF (red fluorescence) and DAPI (blue fluorescence). Representative fluorescence images of the cells; the inset shows higher-magnification images of the areas in the yellow boxes, as indicated. The scale bar is 100 μm. **E** Fluorescence images of BODIPY-THIF in HCT116 cells were quantitatively analyzed with ImageJ software. **F** Colocalization of STAT1 and BODIPY-THIF in CRC cells. HCT116 cells were either treated with BODIPY-THIF (50 μM) alone for 12 h or pretreated with fludarabine (10 μM) for 30 min prior to BODIPY-THIF treatment and subjected to immunofluorescence staining for BODIPY-THIF (red fluorescence), STAT1 (Alexa Fluor 488, green fluorescence) and DAPI (blue fluorescence). Representative fluorescence images of the cells; the inset shows higher-magnification images of the areas in the yellow boxes, as indicated. Merged images are shown. The colocalization signal is colored yellow. Scale bar: 50 μm. **G** The colocalization of BODIPY-THIF and STAT1 in HCT116 cells was quantitatively analyzed with ImageJ software. ***p < 0.001

Molecular docking analysis was performed to investigate the interactions between THIF and the STAT1 protein, and we discovered that THIF might bind to the STAT1 protein (Additional file 1: Fig. S3G). Computational analysis of ligand-receptor interactions showed that THIF docked into the SH2 domain of the STAT1 protein (Additional file 1: Fig. S3H, I). To explain the molecular mechanism of action of anti-STAT1 compounds, we investigated whether THIF could interact directly with STAT1 protein by using SPR biosensor technology. The analysis of sensorgrams by the Biaevaluation evaluation software indicates not only a direct interaction between STAT1 and THIF, but also the presence of more potential sites of their interaction on STAT1 protein (Fig. 2G). To investigate whether THIF affects the STAT1-STAT1 interaction, we then examined the STAT1-STAT1 interaction in the presence and absence of THIF. Using IP analysis, we found that THIF treatment decreased the interaction between phosphorylated STAT1 and STAT1 (Fig. 3A). SHP2 is a well-known STAT1 phosphatase [32]. THIF treatment increased the interaction between phosphorylated SHP2 and STAT1 (Fig. 3A). Deletion of the STAT1 SH2 domain abolished the THIF-mediated effects on the STAT1-SHP2 interaction (Fig. 3B). These results suggest that the STAT1 SH2 domain is essential for the anti-STAT1 activity of THIF.

Fluorescently labeled THIF (BODIPY-THIF) was first designed and synthesized (Additional file 1: Fig. S4; Fig. 3C). An in vitro cellular distribution assay showed that BODIPY-THIF labeled nearly all HCT116 cells after incubation for 12 h (Fig. 3D, E). Immunofluorescence staining further showed that BODIPY-THIF colocalized with the STAT1 protein (Fig. 3F, G). The specificity of BODIPY-THIF binding was investigated by pretreating cells with the published STAT1 inhibitor fludarabine (Fig. 3F, G). This evidence indicates that BODIPY-THIF binds to the STAT1 protein in CRC cells.

### STAT1 enhances colorectal cancer stemness, while this effect is abolished by BODIPY-THIF

**Fig. 4:**
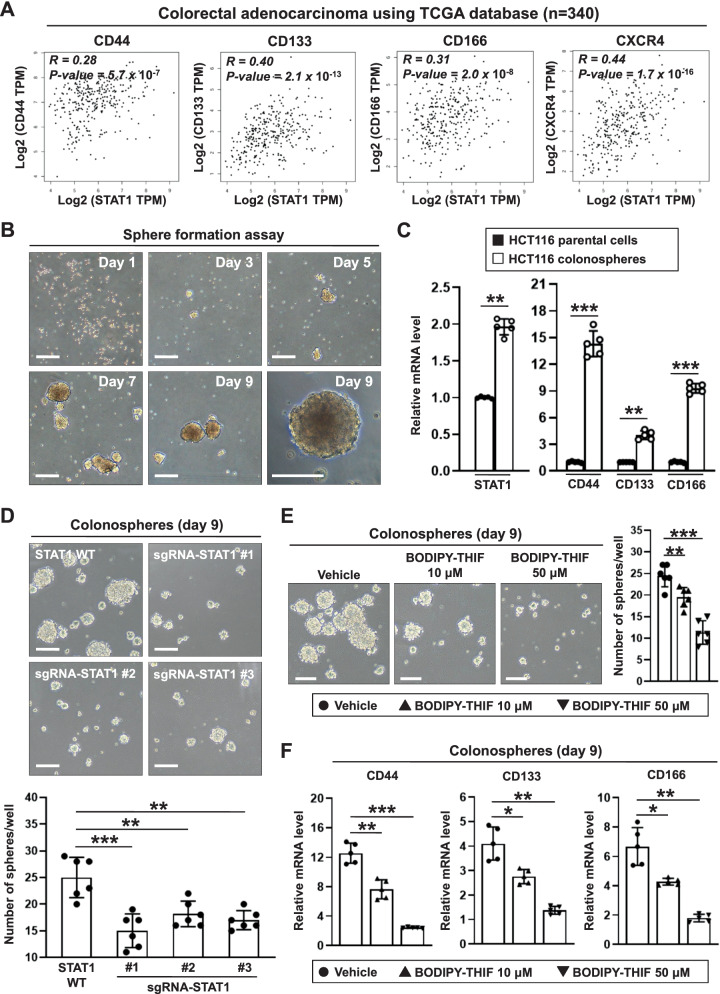
The pharmacological treatment with BODIPY-THIF or ablation of STAT1 via a CRISPR/Cas9-based strategy reduces stemness in colorectal cancer. **A** Correlations between STAT1 and colorectal cancer stem cell (CSC) markers in patients with colorectal adenocarcinoma using TCGA database (https://gdc.cancer.gov/). **B** The expansion of colonospheres was evaluated on days 1, 3, 5, 7 and 9. HCT116 cell morphology was monitored by microscopy. Scale bars: 100 μm. Single-cell suspensions of cells were cultured under bFGF ( +), EGF ( +) and serum-free conditions, allowing them to grow as colonospheres for 9 days. **C** The mRNA expression levels of stemness genes were measured by qPCR. mRNA expression was normalized to GAPDH expression. **p < 0.01; ***p < 0.001. **D** Role of STAT1 in stemness in CRC cells. STAT1 KO HCT116 cells were generated by targeting the STAT1 gene using the CRISPR/Cas9 strategy. STAT1 WT and STAT1 KO cells were cultured in an ultra-low-attachment 6-well plate for 9 days. Cell morphology was monitored by microscopy (upper panel). Scale bars: 100 μm. The number of colonospheres per well was calculated (lower panel). **p < 0.01; ***p < 0.001. **E** HCT116 colonospheres were dissociated into single cells and plated at a density of 5 × 10^4^ cells/well in a 6-well ultra-low-attachment plate. Cells were treated with 10 or 50 μM BODIPY-THIF for 48 h. Cell morphology was monitored by microscopy (left panel). Scale bars: 100 μm. The number of colonospheres was calculated (right panel). **p < 0.01; ***p < 0.001. **F** HCT116 colonospheres were dissociated into single cells and plated at a density of 5 × 10^4^ cells/well in a 6-well ultra-low-attachment plate. Cells were treated with 10 or 50 μM BODIPY-THIF for 48 h. The mRNA expression levels of stemness genes were measured by qPCR. mRNA expression was normalized to GAPDH expression. *p < 0.05; **p < 0.01; ***p < 0.001

Cancer stem cells (CSCs) play an important role in the maintenance, recurrence, metastasis, and drug resistance of CRC, and CD44, CD133, CD166, and CXCR4 are putative surface markers of colorectal CSCs [33–35]. Using The Cancer Genome Atlas (TCGA) database, we found a positive correlation between colorectal CSC-associated markers and STAT1 expression in patients with colon adenocarcinoma (Fig. 4A). In addition, positive correlations between the expression of common CSC-related genes such as NOTCH1, NOTCH2, WNT1, and β-catenin and STAT1 were found (Additional file 1: Fig. S5A). Positive correlations between the expression of CSC markers and STAT1 were also found in human colon tissues using the Genotype-Tissue Expression (GTEx) database (Additional file 1: Fig. S5B).

To study whether STAT1 plays a role in stemness, a colonosphere assay was performed (Fig. 4B). We found that STAT1 was upregulated in colonospheres with stem cell properties compared to the parental cells (Fig. 4C). The formation of colonospheres was inhibited by STAT1 KO (Fig. 4D). BODIPY-THIF or THIF treatment suppressed the formation of colonospheres and inhibited the expression of the stemness markers CD44, CD133, and CD166 (Fig. 4E, F; Additional file 1: Fig. S5C, D).

### STAT1 enhances angiogenesis in colorectal cancer, and BODIPY-THIF shows antiangiogenic activity

**Fig. 5:**
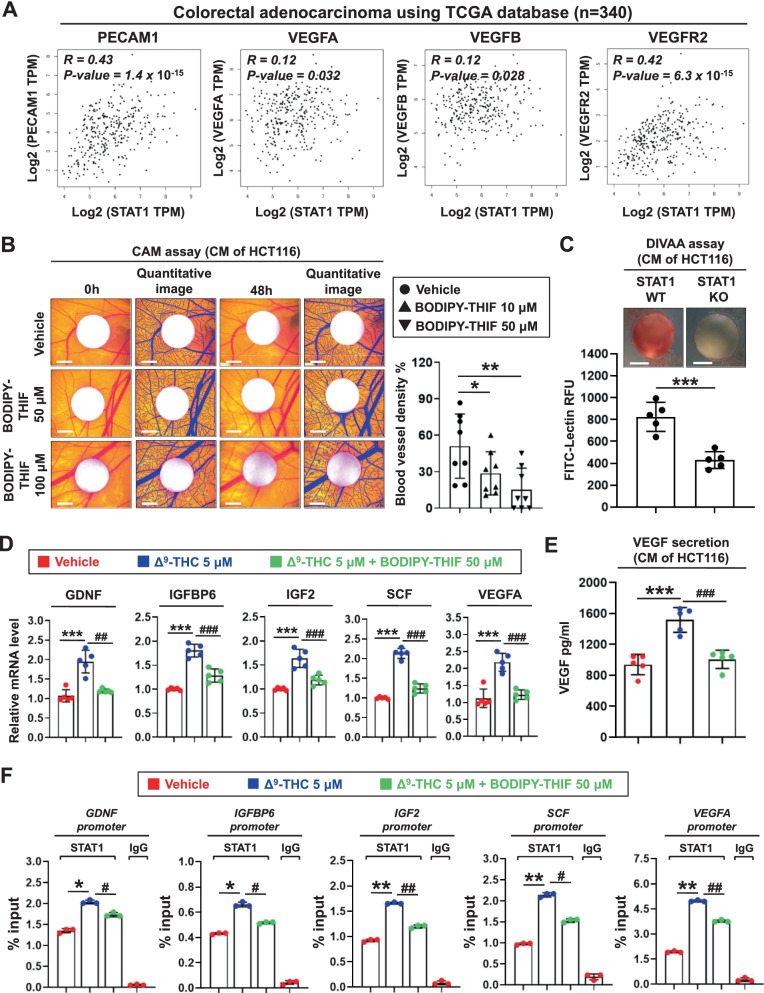
The pharmacological treatment with BODIPY-THIF reduces angiogenesis in colorectal cancer in vitro and in vivo. **A** Correlations between STAT1 and angiogenesis-related markers in patients with colorectal adenocarcinoma using TCGA database. **B** BODIPY-THIF inhibits angiogenesis in vivo. HCT116 cells were treated with vehicle (control) or BODIPY-THIF (50 μM or 100 μM) for 48 h, and CM was collected for the CAM assay. Representative images show the appearance of blood vessels in CAM models (left panel). Images were quantitatively analyzed with WimCAM image analysis software (right panel). *p < 0.05; **p < 0.01. **C** STAT1 WT and STAT1 KO HCT116 cells were cultured for 48 h, and CM was collected for DIVAA. Paired angioreactors (N = 5) were recovered 14 days after implantation. Images of DIVAA angioreactors are shown (upper panel). Quantification of directed in vivo angiogenesis was assessed by FITC-dextran injection (lower panel). ***p < 0.001. **D** Effect of BODIPY-THIF on angiogenesis in CRC cells. HCT116 cells were treated with 5 μM Δ^9^-THC or 5 μM Δ^9^-THC plus 50 μM BODIPY-THIF for 48 h. The mRNA expression levels of the five angiogenesis-related genes were quantified by qPCR and normalized to that of GAPDH. ***p < 0.001 versus vehicle; ^##^*p* < 0.01 versus Δ^9^-THC; ^###^*p* < 0.001 versus Δ^9^-THC. **E** BODIPY-THIF reduces the release of VEGF from CRC cells after Δ^9^-THC treatment. HCT116 cells were treated with 5 μM Δ^9^-THC or 5 μM Δ^9^-THC plus 50 μM BODIPY-THIF for 48 h, and the VEGF concentration in CM was measured by ELISA. ***p < 0.001 versus vehicle; ^###^*p* < 0.001 versus Δ^9^-THC. **F** Effect of BODIPY-THIF on the binding of STAT1 to the promoters of angiogenesis-related growth factors in CRC cells. HCT116 cells were treated with 5 μM Δ^9^-THC or 5 μM Δ^9^-THC plus 50 μM BODIPY-THIF for 6 h, and the anti-STAT1 antibody was used for ChIP-qPCR to measure the binding of STAT1 to the promoters. Data were analyzed and are plotted as percent (%) of input DNA. *p < 0.05 versus vehicle; **p < 0.01 versus vehicle; ^#^*p* < 0.05 versus Δ^9^-THC; ^##^*p* < 0.01 versus Δ^9^-THC

Angiogenesis is a hallmark of cancer [36, 37]. Using the TCGA database, we found positive correlations between the expression of angiogenesis-related markers and STAT1 in patients with colon adenocarcinoma (Fig. 5A). Positive correlations between the expression of angiogenesis-related markers and STAT1 were also found in human colon tissues using the GTEx database (Additional file 1: Fig. S6A).

To study whether BODIPY-THIF inhibits CRC angiogenesis in vivo, a CAM assay and a DIVAA were used (Additional file 1: Fig. S6B, C). BODIPY-THIF was found to inhibit angiogenic abilities in the CAM assay (Fig. 5B). DIVAA showed that STAT1 KO reduced in vivo angiogenesis, and Q-PCR and Western blot analysis confirmed the KO efficacy (Fig. 5C; Additional file 1: Fig. S6D). In our previous study, we found that Δ^9^-THC induced angiogenesis in CRC through STAT1 activation [31]. We next studied the effect of BODIPY-THIF on Δ^9^-THC-induced angiogenesis. The levels of a variety of angiogenic growth factors, such as GDNF, IGFBP6, IGF2, SCF, and VEGFA, were increased in the CM of Δ^9^-THC-treated HCT116 cells, while these effects were significantly reduced by BODIPY-THIF treatment (Fig. 5D). In addition, Δ^9^-THC-induced secretion of VEGF was abolished by BODIPY-THIF (Fig. 5E). The ChIP-qPCR assay revealed that Δ^9^-THC induced binding of STAT1 to the promoter regions of angiogenic growth factors, while these effects were reversed by BODIPY-THIF (Fig. 5F). THIF showed similar effects to those of BODIPY-THIF on angiogenesis (Additional file 1: Fig. S6E, F).

### BODIPY-THIF reverses Δ^9^-THC-induced tumor growth in the mouse model of AOM/DSS-induced colorectal cancer

**Fig. 6:**
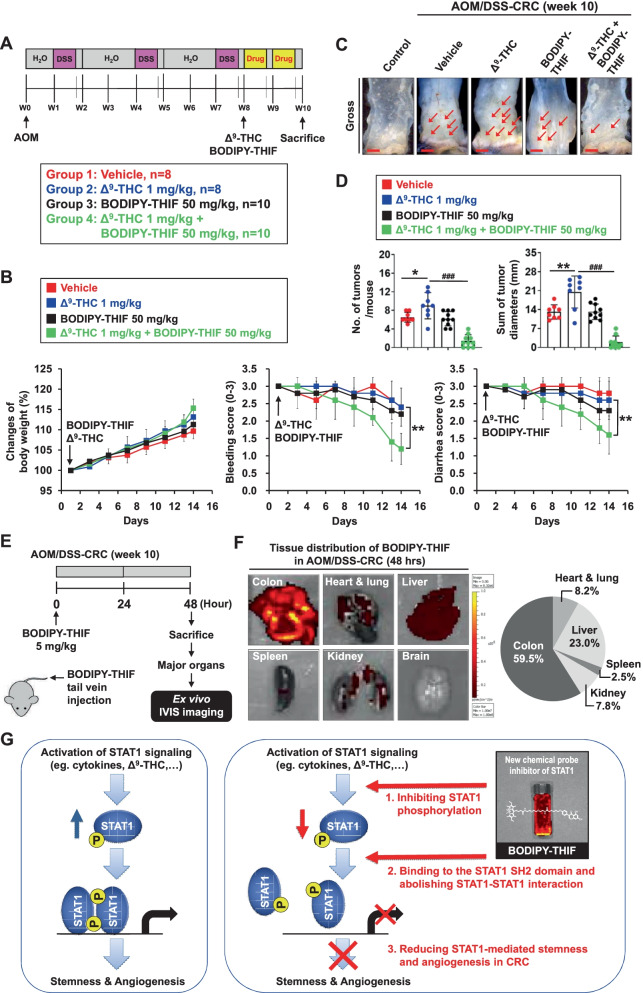
Effect of BODIPY-THIF on tumor growth in the mouse model of AOM/DSS-induced colorectal cancer. AOM/DSS-treated mice were randomly divided into four groups. Mice were treated with vehicle (control), Δ^9^-THC (1 mg/kg, intraperitoneally), BODIPY-THIF (50 mg/kg, orally), or Δ^9^-THC (1 mg/kg, intraperitoneally) plus BODIPY-THIF (50 mg/kg, orally) five days a week for two weeks after AOM/DSS treatment. **A** Schematic overview of the experimental design. **B** Changes in body weight (left panel), clinical bleeding scores (middle panel), and clinical diarrhea scores (right panel) during treatment are shown. **p < 0.01 versus Δ^9^-THC. **C** The therapeutic effect of BODIPY-THIF on AOM/DSS-induced CRC. Gross images of terminal colons are shown, and the red arrowhead indicates macroscopic polyps. Scale bars: 1 mm. **D** The number and size of tumors were plotted. *p < 0.05 and **p < 0.01 versus vehicle; ^###^*p* < 0.001 versus Δ^9^-THC. **E** and **F** AOM/DSS-CRC mice at week 10 were injected with BODIPY-THIF (5 mg/kg, intravenously). After 48 h, the mice were sacrificed, and all major organs were collected for ex vivo IVIS imaging. **E** Schematic overview of the experimental design. **F** The distribution of BODIPY-THIF in AOM/DSS mouse models. BODIPY-THIF accumulated in the colon and liver but was minimally detected in the heart, lung and brain (left panel). Fluorescence images were quantitatively analyzed using the IVIS imaging system (right panel). **G** Model for the regulation of STAT1 activation and antitumor effects of the STAT1 chemical probe inhibitor BODIPY-THIF. Activation of STAT1 signaling plays a critical role in stemness and angiogenesis in CRC. Genetic inhibition of STAT1 can attenuate these effects in CRC. THIF, a natural flavonoid abundant in soybeans, can attenuate STAT1-mediated effects in vitro and in vivo. THIF not only inhibits the phosphorylation of STAT1 but also binds to the STAT1 SH2 domain and abolishes the STAT1-STAT1 interaction in CRC. BODIPY-THIF is a new chemical probe inhibitor of STAT1 that reduces stemness and angiogenesis in CRC

As BODIPY-THIF can inhibit stemness and angiogenesis in CRC, the anti-CRC effect of BODIPY-THIF was further evaluated in a mouse model of AOM/DSS-induced CRC (Fig. 6A). Symptom parameters, such as body weight loss, diarrhea, and rectal bleeding, were observed in mice after AOM/DSS treatment, and BODIPY-THIF reduced the occurrence of these symptoms (Additional file 1: Fig. S7A-C; Fig. 6B). BODIPY-THIF did not affect colon length in AOM/DSS-treated mice (Additional file 1: Fig. S7D). Δ^9^-THC increased the number and size of colon tumors in AOM/DSS model mice, while these effects were reversed by BODIPY-THIF treatment (Additional file 1: Fig. S7E; Fig. 6C, D). In addition to the AOM-DSS model, the effect of BODIPY-THIF was examined in a CRC xenograft mouse model (Additional file 1: Fig. S7F). Δ^9^-THC enhanced CRC tumor growth, while these effects were abolished by BODIPY-THIF (Additional file 1: Fig. S7G, H).

To study the in vivo biodistribution of BODIPY-THIF, AOM/DSS-treated mice at week 10 of model establishment were intravenously injected with BODIPY-THIF (Fig. 6E). After 48 h, the major organs were isolated. Ex vivo IVIS imaging showed accumulation of BODIPY-THIF in the colon and liver and minimal detection of BODIPY-THIF in other major organs (Fig. 6F).

## Discussion

STAT1 plays an essential role in the immune response and protects against pathogen infections; thus, STAT1 is generally considered a tumor suppressor. [13, 14]. Take CRC for example. STAT1 has been shown to inhibit the growth of CRC through the down-regulation of miR-181a [38]. In addition, low expression levels of STAT1 were observed in colon epithelial cells under oncogenic transformation [39]. It has also been reported that intestinal fibroblasts induce STAT1 activation in CRC cells and inhibit the growth of CRC [40]. These reports indicate the tumor suppressive roles of STAT1 in CRC. In contrast, STAT1 has been shown to induce the proliferation of CRC through the upregulaiton of the STAT1-CCL5 axis [41]. It has also been reported that STAT1 promotes KRAS colon tumor growth by downregulating programmed cell death protein 4 [42]. Chronic stimulation of the death receptor CD95 has been shown to induce stemness of CRC through the activation of the STAT1-meidated response [43]. For some oncogenes, such as CUG2, oncogenic signaling pathway-mediated STAT1 activation significantly induced the metastatic and drug-resistance properties of CRC [44]. These reports indicate the oncogenic roles of STAT1 in CRC. In this study, overexpression of STAT1 was found in patients with CRC and in mouse models of AOM/DSS-induced CRC and metastatic CRC. We found that STAT1 played an important role in stemness and angiogenesis in CRC and that these effects were reversed by the chemical probe inhibitor BODIPY-THIF or ablation of STAT1.

STAT1 plays a pivotal role in both the adaptive and innate host immune responses [45]. In response to IFN, STAT1 mediates CD4 and CD8 T-cell activation and differentiation toward a T helper 1 immune response [46]. STAT1 also plays an important role in B cell differentiation [47]. In addition to adaptive immunity, STAT1 has distinct roles in innate host immune responses. STAT1 is a critical mediator of macrophage polarization [48]. Therefore, blocking STAT1 in vivo may weaken the host immune system and cause adverse effects for patients. For example, STAT1-deficent mice are more susceptible to viral infection than wild-type mice [49]. Stat1-deficent mice develop aggravated Th17-mediated autoimmune diseases [50]. In this study, BODIPY-THIF treatment significantly reduced tumor growth in AOM/DSS-CRC mice. Studies have demonstrated that massive infiltration of macrophages and neutrophils into the lamina propria and submucosa in DSS-mediated acute colitis and in the progression of colitis-associated colon carcinogenesis in mice [51]. To investigate whether immune cells are affected by BODIPY-THIF, IHC staining was performed in colorectal tumor section stained with the pan-macrophage marker F4/80 or activated macrophage marker CD68 [52, 53]. The infiltrations of macrophages were abundant in AOM-DSS CRC tumors and were blocked by BODIPY-THIF treatment (Additional file 1: Fig. S8A, B). These results suggest that mouse immune system may be influenced by BODIPY-THIF treatment. A long-term follow-up of BODIPY-THIF treatment in immune response needs further investigation.

Members of the STAT family have been implicated in cancer development, progression, metastasis, survival and chemotherapeutic resistance [12]. Among the STAT family members, STAT3 is the most well-studied member. STAT3 is an attractive molecular target for the development of new anticancer therapeutics [54–56]. Similar to many other transcription factors, STAT3 is difficult to target directly. Therefore, indirect targeting strategies such as JAK inhibitors have led to the development of STAT3 inhibitors [57, 58]. However, JAK phosphorylates multiple protein substrates in addition to STAT3 [59]. Thus, JAK inhibitors may cause unwanted off-target effects. A more specific strategy to inhibit STAT signaling is therefore urgently needed. In this study, we found that THIF did not affect the phosphorylation of JAK2, confirming the selectivity of THIF in targeting STAT1 signaling without influencing JAK signaling. Binding to the SH2 domain is highly specific and a critical step in determining the specificity of receptor-mediated STAT activation [60]. THIF directly bound to the STAT1 SH2 domain and abolished the STAT1-STAT1 interaction. Recently, proteolysis-targeting chimeras (PROTAC) have been developed as a useful technology for the development of therapeutics that induce targeted protein degradation [61, 62]. A PROTAC compound is a bifunctional small molecule that can target proteins for ubiquitylation by an E3 ubiquitin ligase and subsequent degradation by the proteasome. STAT3 PROTAC degraders have been reported to downregulate STAT3 and showed therapeutic effects in leukemia and lymphoma cells [63, 64]. STAT1 PROTAC degraders are currently under investigation (data not shown). Our results indicated that STAT1 PROTAC degraders could induce STAT1 protein degradation and inhibit CRC cell growth.

Fluorescent chemical probes are of use in the development of cancer imaging and play roles in the study of cancer biology and metabolism [26, 27]. Fluorescein, BODIPY, rhodamine and cyanine are promising organic dyes for various biological applications [65]. Among these molecules, BODIPY has been considered fluorescent molecule used in applications ranging from fundamental biological research to clinical diagnostics and theranostics over the past decade due to its excellent photostable and photophysical properties as well as its relative insensitivity in the physiological pH range [66]. In particular, the properties of BODIPYs can be easily turned by chemical modification on the dipyrromethene core, providing a versatile platform for further functionalization of all aspects of desirable photophysical and theranostic properties [67]. In this study, we first designed and synthesized the chemical probe inhibitor BODIPY-THIF to target STAT1. BODIPY-THIF showed high penetration into CRC cells and tumors. Direct evidence of BODIPY-THIF binding STAT1 was validated by in vitro and in vivo studies. In vivo binding was also investigated using intravenously injected BODIPY-THIF in mice with AOM/DSS-induced CRC, and BODIPY-THIF was shown to accumulate mainly in the colon. These results suggest that BODIPY-THIF is a new chemical probe inhibitor of STAT1 and can be a potential tool for CRC therapy as well as cancer cell imaging. Similar approaches were found by other research groups, in which BODIPY-conjugated probes were developed for cancer cell imaging and therapeutic use [68, 69]. For example, BODIPY-Taxol showed high accumulation and extensive distribution throughout breast cancer tumor spheroid models [70]. Cisplatin conjugated to BODIPY (Pt-BODIPY) was found to be more cytotoxic than unconjugated cisplatin in cisplatin-resistant cervical cancer cells [71].

Medical marijuana has been approved by the FDA for treating chemotherapy-induced nausea and vomiting [72]. However, relatively little is known about its direct effects on tumor cells and the tumor microenvironment. To date, two synthetic cannabis drugs have been approved by the FDA for treating chemotherapy-induced nausea and vomiting as well as HIV and anorexia. These drugs are Marinol® (dronabinol), Syndros® (dronabinol), and Cesamet® (nabilone). The active ingredient in synthetic cannabis drugs is Δ^9^-THC, the main mind-altering compound in marijuana [72]. Our previous study showed that Δ^9^-THC can induce angiogenesis and tumor progression in CRC through activation of STAT1 [31]. In this study, we used a high-throughput virtual screening strategy and identified THIF, a flavonoid abundant in soybeans, as a putative STAT1 inhibitor. Five angiogenic growth factors, GDNF, IGFBP6, IGF2, SCF, and VEGFA, were upregulated in Δ^9^-THC-treated CRC cells, while these effects were reversed by BODIPY-THIF treatment. Δ^9^-THC-induced binding of STAT1 to the promoters of the five growth factors and angiogenesis were also reversed by BODIPY-THIF. In mouse models of AOM/DSS-induced CRC, the number and size of colon tumors were increased after Δ^9^-THC treatment. This finding was consistent with our previous findings in CRC xenograft and tail vein injection mouse models, and BODIPY-THIF treatment can significantly inhibit tumor growth in AOM/DSS models. We hypothesize that clinical cotreatment with THIF and Δ^9^-THC would allow beneficial effects of Δ^9^-THC, such as appetite stimulation and sedation, while protecting against adverse effects on tumor cells and the tumor microenvironment in patients with CRC.

Multitarget effects are a common problem of small molecule compounds, including flavonoid derivatives [73]. For example, flavonoids have been reported to bind to estrogen receptors due to the similarity of their chemical structure to that of estrogen [74]. Flavonoids have also been found to suppress the kinase activity of EGFR, ERK1/2, RSK2, MKK4, and Cot in an ATP-competitive manner [75]. To evaluate whether THIF would bind to other targets, ligand-based virtual screening for target prediction was performed using the SwissTargetPrediction database [76]. The results showed that THIF may target other proteins, such as thromboxane-A synthase, monoamine oxidase A, EGFR, and estrogen receptor (Additional file 1: Table S2). THIF exerts an anti-CRC effect by targeting STAT1, but we cannot exclude the possibility that THIF functions via other additional mechanisms independent of the STAT1-mediated pathway. Epidemiological studies have found a lower incidence of several cancer types, such as CRC, in populations in Asian countries than in those in the Western world [77]. THIF is the main isoflavone contained in soy-based foods that are regularly consumed by people in Asian countries [78]. Soy isoflavone is considered a safe food supplement [79]. Despite the multitarget effects of THIF, it should be relatively safe and is worth evaluating as a lead compound for further optimization.

## Conclusions

Our findings indicate that STAT1 plays a vital role in stemness and angiogenesis in CRC. BODIPY-THIF is a new chemical probe inhibitor of STAT1 that reduces stemness and angiogenesis in CRC. BODIPY-THIF can be a promising tool for CRC therapy as well as cancer cell imaging (Fig. 6G).

## Supplementary Information

**Additional file 1: Figure S1.** Expressions of STAT1 in primary and metastatic colorectal cancer. (A) A summary of the each individual microarray datasets from different GEO dataset (https://www.ncbi.nlm.nih.gov/gds). (B) The GDS4382 dataset containing microarray data for colorectal tumor tissues and adjacent normal tissues was obtained from the NCBI GEO database. The expression levels of STAT1 are shown. *p< 0.05; ***p< 0.001. (C) The GDS3501 dataset containing microarray data for patients with metastatic CRC was obtained from the NCBI GEO database. The expression levels of STAT1 are shown. (D) Western blot analysis and Q-PCR analysis for STAT1 expressions in normal human colon epithelium cell line (FHC) and CRC cell lines (HCT116, HT29, RKO, and SW480). Total cell lysates were prepared, and Western blot analysis was performed (upper panel). Q-PCR analyzed the mRNA expressions of STAT1, which were normalized to GAPDH (lower panel). **p< 0.01 versus FHC; ***p< 0.001 versus FHC. (E) Tumor sections from the AOM/DSS-induced CRC mouse models were counterstained with H&E, and high-magnification images of the yellow-boxed area are shown. Scale bars: 250 μm. (F) Schematic overview of the experimental design (left panel). Seven-week-old male NOD/SCID mice were intravenously injected with HCT116 cells (1 × 106) through the tail vein. Mice were sacrificed at 3 weeks, and lung segments were fixed by formalin. Lung sections from the tail vein-injected mouse models were counterstained with H&E, and high-magnification images of the yellow-boxed area are shown (right panel). Scale bars: 250 μm. **Figure S2.** Generation of STAT1 knockout colorectal cancer cells using CRISPR/Cas9 system. (A) Schematic overview of the CRISPR/Cas9 system in CRC cells. CRISPR plasmids expressing Cas9 and STAT1 sgRNAs were transfected into HCT116 cells for 12 hours. Cells were treated with puromycin (0.5 μg/ml) for 14 days. Single clones were picked according to GFP expression and expanded to generate monoclonal cell colonies. (B) Successful transfection of CRISPR/Cas9 KO plasmids could be visually confirmed by detection of the GFP. The white arrowhead indicates the GFP-positive HCT116 cells. Scale bar: 100 μm. (C) GFP-positive HCT116 cells were calculated. (D) The STAT1 mRNA levels in HCT116 cells treated with STAT1 sgRNAs versus control were quantified by qPCR analysis. The mRNA expression was normalized to GAPDH. N.D., not detected for 40 cycles by qPCR. (E) Role of STAT1 on cell viability in HCT116 cells. STAT1 WT cells and STAT1 KO cells were incubated, and cell viability was measured at 48 hours by the MTT assay. ***p < 0.001. (F) Changes of the body weights in HCT116 xenograft mouse models are shown. **Figure S3.** THIF can directly bind to the STAT1 protein. (A) Effect of THIF on STAT1 phosphorylation in CRC cells. HCT116 cells were treated with STAT1 activator (500 ng/ml IFN-γ) or STAT1 activator plus THIF (10 μM or 50 μM) for 12 hours. Total protein extract was prepared and Western blot analysis was performed using indicated antibodies (upper panel). Western blot images were quantified by ImageJ software (lower panel). (B) Effect of THIF on STAT1 phosphorylation in CRC cells. HCT116 cells were treated with 5 μM Δ9-THC, Δ9-THC plus 10 μM THIF or Δ9-THC plus 10 μM fludarabine for 12 hours. Protein extracts from the nuclear and cytoplasmic fractions were prepared. Western blot analysis was performed using the indicated antibodies. Western blot images were quantitatively analyzed with ImageJ software. (C and D) HCT116 cells were treated with 5 μM Δ9-THC, 50 μM THIF or their combination for 48 hours. (C) Protein extract was prepared and Western blot analysis was performed using indicated antibodies (upper panel). Western blot images were quantified by ImageJ software (lower panel). (D) qPCR analysis was performed. The mRNA expression was normalized to GAPDH. N.S., no significance. (E) Dose-response effect of THIF on STAT1 activity. The luciferase activity of STAT1 was measured by the STAT1 reporter assay in 293T cells. Cells were treated with increasing concentrations of THIF for 12 hours. The 50% of inhibition concentration (IC50) of luciferase activity is calculated by SigmaPlot software. (F) HCT116 cells were treated with indicated concentrations of THIF for 48 hours. Protein extract was prepared and Western blot analysis was performed using indicated antibodies. (G-H) Conformations of THIF docked in binding sites on the STAT1 protein. (G) Docking models of THIF in the STAT1 crystal structure (PDB: 1YVL). The predicted binding modes of THIF and STAT1 are shown. The white circle indicates the binding site of THIF. (H) The binding interactions between THIF and amino acid residues in STAT1 protein are shown. (I) The overlapping binding sites of four predicted binding modes of THIF with the STAT1 SH2 domain are highlighted in blue. **Figure S4.** A synthesis plan of fluorescently labeled STAT1 probe inhibitor. Reactions are magnetically stirred and monitored by thin-layer chromatography on silica gel. Flash chromatography is performed on silica gel of 60-200 μm particle size. Yields are reported for spectroscopically pure compounds. Melting points are recorded on a Fargo MP-2D melting point apparatus. 1H, 13C, and 31P NMR spectra are recorded on Bruker AV 600 (600 MHz), Bruker AV 500 (500 MHz) and Bruker AVIII 17 400 (400 MHz) spectrometers. Chemical shifts are given in δ values relative to tetramethylsilane (δH = 0); coupling constants J are given in Hz. Internal standards are CDCl3 (δH = 7.24), CD3OD (δH = 3.31) or DMSO-d6 (δH = 2.49) for 1H NMR spectra and CDCl3 (δC = 77.0, central line of triplet), CD3OD (δC = 49.0, central line of septet) or DMSO-d6 (δC = 39.5, central line of septet) for 13C NMR spectra. The splitting patterns are reported as s (singlet), d (doublet), t (triplet), q (quartet), m (multiplet), br (broad), and dd (double of doublets). High-resolution electrospray ionization and fast atom bombardment mass spectra were recorded on a JMS-T100LP AccuTOF LC-plus 4G mass spectrometer. **Figure S5.** Effect of THIF on cancer stemness. (A) Correlations between STAT1 and common cancer stem cell (CSC) markers are shown in patients with colorectal adenocarcinoma using TCGA database (https://gdc.cancer.gov/). (B) Correlations between STAT1 and CSC markers are shown in human colon tissues using the GTEx database (https://www.gtexportal.org/home/). (C and D) HCT116 colonospheres were dissociated into single cells and plated at a density of 5 × 104 cells/well in a 6-well Ultra-Low plate. Cells were treated with 10 or 50 μM THIF for 48 hours. (C) Cell morphology was monitored by microscopy (upper panel). Scale bars: 100 μm. The number of colospheres was calculated (lower panel). **p< 0.01; ***p< 0.001. (D) The mRNA expression of stemness genes was measured by qPCR. The mRNA expression was normalized to GAPDH. *p< 0.05; **p< 0.01; ***p< 0.001. **Figure S6.** Effect of THIF on angiogenesis in colorectal cancer in vitro and in vivo. (A) Correlations between STAT1 and angiogenesis-related markers are shown in human colon tissues using the GTEx database. (B) Schematic overview of the experimental design for in vivo angiogenesis assays. (C) HCT116 cells were treated with vehicle (control) or BODIPY-THIF (50 μM or 100 μM) for 48 hours, and the CM was collected for the chick chorioallantoic membrane (CAM) assay. Sterilized filter-paper disks were used as a carrier for CM of HCT116 cells. Images were quantified by WimCAM image analysis software. (D) Validation of STAT1 expression by Q-PCR and western blot analysis in STAT1 KO cells. The STAT1 protein levels and mRNA levels in HCT116 cells treated with STAT1 sgRNAs versus control were quantified by Western blot analysis and Q-PCR analysis. Protein extract was prepared and Western blot analysis was performed using indicated antibodies (upper panel). mRNA expression was normalized to GAPDH (lower panel). N.D., not detected for 40 cycles by Q-PCR. Data are representative of three independent experiments and values are expressed in mean ± SD. (E) Effect of THIF on Δ9-THC-induced angiogenesis in CRC cells. HCT116 cells were treated with 5 μM Δ9-THC or 5 μM Δ9-THC plus 50 μM THIF for 48 hours. The mRNA expressions of the five angiogenesis-related genes were quantified by qPCR analysis and normalized to GAPDH. ***p< 0.001 versus vehicle; ##p < 0.01 versus Δ9-THC; ###p < 0.001 versus Δ9-THC. (F) THIF reduces the release of VEGF from CRC cells after Δ9-THC treatment. HCT116 cells were treated with 5 μM Δ9-THC or 5 μM Δ9-THC plus 50 μM THIF for 48 hours, and VEGF concentration in conditioned medium (CM) was measured by ELISA. ***p< 0.001 versus vehicle; ###p < 0.001 versus Δ9-THC. **Figure S7.** Effects of BODIPY-THIF on tumor growth in AOM/DSS-induced colorectal cancer mouse models. CRC was induced by intraperitoneal injection of AOM (12.5 mg/kg) in conjunction with the DSS stimulus. Mice were maintained with a regular diet and drinking water for 7 days and then subjecting to 3 cycles of DSS treatment, with each cycle consisting of the administration of 3.5% DSS for 5 days followed by a 14-day recovery period with regular water. (A-C) Colitis symptom in AOM/DSS mouse models during the CRC induction and recovery phases. (A) Changes in body weights are shown. (B) Clinical bleeding scores are shown. (C) Clinical diarrhea scores are shown. (D) Representative whole colons (upper panel) and colon length (lower panel) are shown. Scale bar, 2 cm. ***p< 0.001 versus healthy control. (E) Representative whole colons are depicted, and the yellow arrowhead indicates macroscopic polyps. Scale bars: 5 mm. (F-H) Effect of BODIPY-THIF on colorectal tumor growth in xenograft mouse models. HCT116 cells (1 × 107 cells) were subcutaneously implanted into male nude mice. When the tumor volume reached 100 mm3, mice were treated with vehicle (control), Δ9-THC (1 mg/kg, intraperitoneally), or Δ9-THC (1 mg/kg, intraperitoneally) plus BODIPY-THIF (50 mg/kg, orally) every two days for two weeks. The tumor volume was measured every two days after drug treatment. (F) Schematic overview of the experimental design in the mouse model. (G) The tumor volume was calculated as follows: V = 0.5 × (the length of length) × (the length of width)2. **p< 0.01 versus vehicle; ***p< 0.001 versus vehicle; ###p < 0.001 versus Δ9-THC. (H) Gross pictures of xenograft tumors are shown (upper panel). Scale bar: 1 cm. At the end of the experiment, the excised tumors were weighed (lower panel). ***p< 0.001 versus vehicle; ###p < 0.001 versus Δ9-THC. Figure 8. Effects of STAT1 on immune response in colorectal cancer. AOM/DSS-treated mice were randomly divided into four groups. Mice were treated with vehicle (control), Δ9-THC (1 mg/kg, intraperitoneally), BODIPY-THIF (50 mg/kg, orally), or Δ9-THC (1 mg/kg, intraperitoneally) plus BODIPY-THIF (50 mg/kg, orally) five days a week for two weeks after AOM/DSS treatment. Colon sections were immunostained with anti-F4/80 (A) and anti-CD68 (B) antibodies, and one representative experiment of three was presented. High-magnification images of the areas in the black boxes are shown. Scale bar, 250 μm. **Table S1.** List of antibodies used in this study. **Table S2.** Predicted protein targets of THIF from the SwissTargetPrediction Database.

## Data Availability

All materials are available by the corresponding author.

## Abbreviations

AOM: Azoxymethane
BODIPY-THIF: Fluorescently labeled 4’,5,7- trihydroxyisoflavone probe
CAM: Chick chorioallantoic membrane
CRC: Colorectal cancer
CM: Conditioned medium
CSC: Cancer stem cell
DIVAA: Directed in vivo angiogenesis assay
DMSO: Dimethyl sulfoxide
DSS: Dextran sulfate sodium
GEO: Gene expression omnibus
GFP: Green fluorescent protein
GTEx: Genotype-tissue expression
IFN: Interferon
IC_50_: 50% Of inhibition concentration
IHC: Immunohistochemistry
IP: Immunoprecipitation
JAK: Janus kinase
KO: Knockout
MTT: 3-(4,5-Dimethylthiazol-2-yl)-2,5-diphenyl tetrazolium bromide
PROTAC: Proteolysis-targeting chimeras
SD: Standard deviation
SPR: Surface plasmon resonance
STAT: Signal transducer and activator of transcription
TCGA: The cancer genome atlas
THIF: 4’,5,7-Trihydroxyisoflavone
Δ^9^-THC: Δ^9^-Tetrahydrocannabinol
WT: Wild-type
