# Supplementary material for: A chemical probe inhibitor targeting STAT1 restricts cancer stem cell traits and angiogenesis in colorectal cancer
Source: J Biomed Sci. 2022 Mar 22;29:20. doi: 10.1186/s12929-022-00803-4 (PMC8939146; doi:10.1186/s12929-022-00803-4)
Supplement: Supplementary file 1 — Additional file 1: Figure S1. Expressions of STAT1 in primary and metastatic colorectal cancer. (A) A summary of the each individual microarray datasets from different GEO dataset (https://www.ncbi.nlm.nih.gov/gds). (B) The GDS4382 dataset containing microarray data for colorectal tumor tissues and adjacent normal tissues was obtained from the NCBI GEO database. The expression levels of STAT1 are shown. *p< 0.05; ***p< 0.001. (C) The GDS3501 dataset containing microarray data for patients with metastatic CRC was obtained from the NCBI GEO database. The expression levels of STAT1 are shown. (D) Western blot analysis and Q-PCR analysis for STAT1 expressions in normal human colon epithelium cell line (FHC) and CRC cell lines (HCT116, HT29, RKO, and SW480). Total cell lysates were prepared, and Western blot analysis was performed (upper panel). Q-PCR analyzed the mRNA expressions of STAT1, which were normalized to GAPDH (lower panel). **p< 0.01 versus FHC; ***p< 0.001 versus FHC. (E) Tumor sections from the AOM/DSS-induced CRC mouse models were counterstained with H&E, and high-magnification images of the yellow-boxed area are shown. Scale bars: 250 μm. (F) Schematic overview of the experimental design (left panel). Seven-week-old male NOD/SCID mice were intravenously injected with HCT116 cells (1 × 10^6^) through the tail vein. Mice were sacrificed at 3 weeks, and lung segments were fixed by formalin. Lung sections from the tail vein-injected mouse models were counterstained with H&E, and high-magnification images of the yellow-boxed area are shown (right panel). Scale bars: 250 μm. Figure S2. Generation of STAT1 knockout colorectal cancer cells using CRISPR/Cas9 system. (A) Schematic overview of the CRISPR/Cas9 system in CRC cells. CRISPR plasmids expressing Cas9 and STAT1 sgRNAs were transfected into HCT116 cells for 12 hours. Cells were treated with puromycin (0.5 μg/ml) for 14 days. Single clones were picked according to GFP expression and expanded to generate monocl [file 12929_2022_803_MOESM1_ESM.pdf]

Additional File

A chemical probe inhibitor targeting STAT1 restricts cancer stem cell traits and angiogenesis in colorectal cancer

Pei-Hsuan Chou[1], Cong-Kai Luo[1], Niaz Wali[2,3,4], Wen-Yen Lin[5], Shang-Kok Ng[1], Chun-Hao Wang[6], Mingtao Zhao[7,8,9], Sheng-Wei Lin[10], Pei-Ming Yang[11], Pin-Jung Liu[12], Jiun-Jie Shie[2]*, Tzu-Tang Wei[1,4]*

Inventory of Additional File

Figure S1-S2, related to Figure 1

Figure S3, related to Figure 2

Figure S4, related to Figure 3

Figure S5, related to Figure 4

Figure S6, related to Figure 5

Figure S7, related to Figure 6

Figure S8

Table S1

Table S2

Figure S1

A

GEO Accession	Dataset	Platform	Description and sample size
GSE32323	GDS4382	GPL570	■ RNA samples were extracted from colonic mucosa of CRC patients.<br>■ Primary colorectal cancer paired with non-cancerous tissues (**17 paired samples**).
GSE10961	GDS3501	GPL570	■ RNA samples were extracted from liver metastases of CRC patients.<br>■ Contained synchronous liver metastasis (**10 samples**) and metachronous liver metastasis (**8 samples**).

GPL570, [HG-U133_Plus_2] Affymetrix Human Genome U133 Plus 2.0 Array.

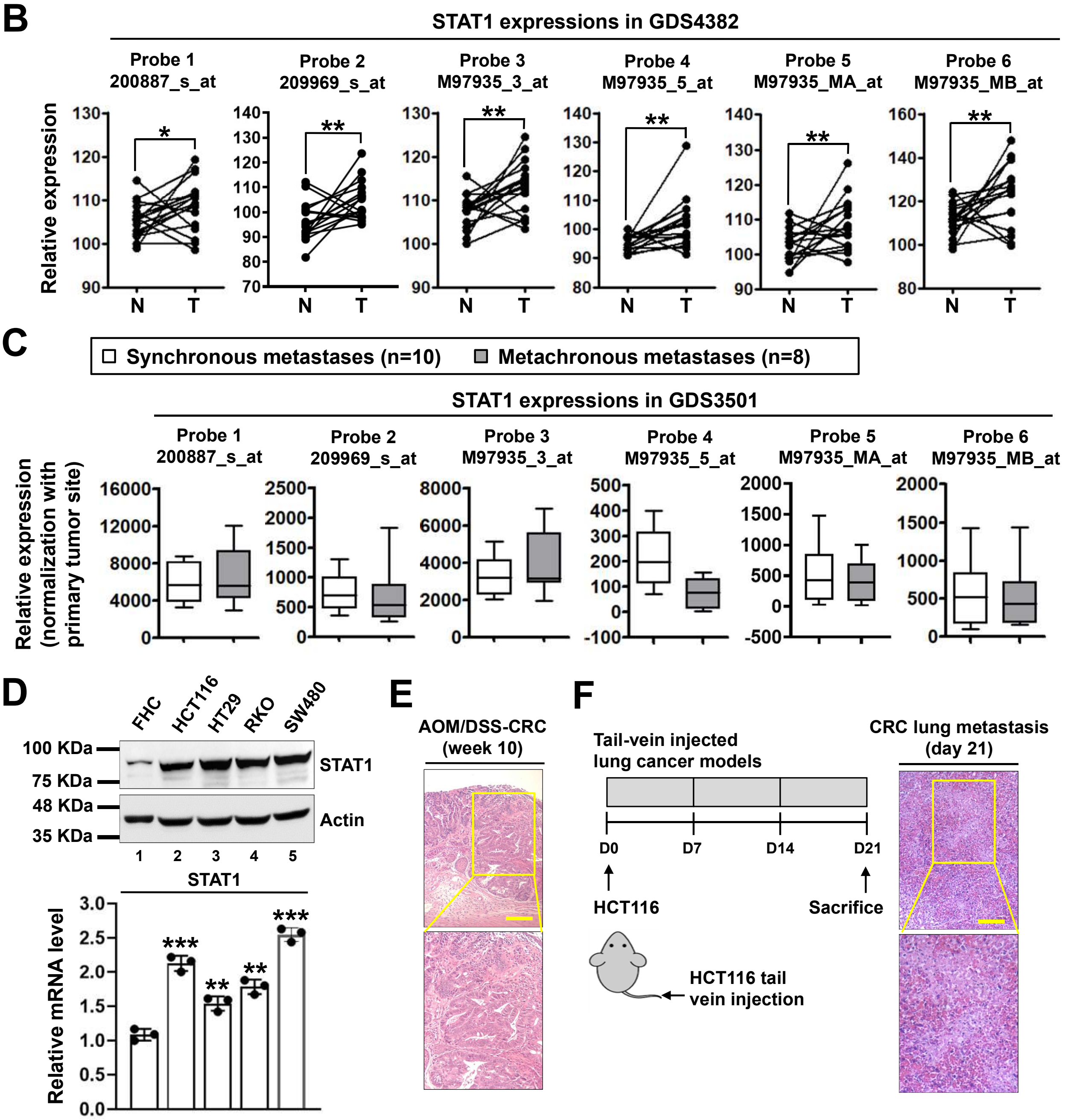

Figure S1. Expressions of STAT1 in primary and metastatic colorectal cancer. (A) A summary of the each individual microarray datasets from different GEO dataset (https://www.ncbi.nlm.nih.gov/gds). **(B)** The GDS4382 dataset containing microarray data for colorectal tumor tissues and adjacent normal tissues was obtained from the NCBI GEO database. The expression levels of STAT1 are shown. *p< 0.05; ***p< 0.001. **(C)** The GDS3501 dataset containing microarray data for patients with metastatic CRC was obtained from the NCBI GEO database. The expression levels of STAT1 are shown. **(D)** Western blot analysis and Q-PCR analysis for STAT1 expressions in normal human colon epithelium cell line (FHC) and CRC cell lines (HCT116, HT29, RKO, and SW480). Total cell lysates were prepared, and Western blot analysis was performed (upper panel). Q-PCR analyzed the mRNA expressions of STAT1, which were normalized to GAPDH (lower panel). **p< 0.01 versus FHC; ***p< 0.001 versus FHC. **(E)** Tumor sections from the AOM/DSS-induced CRC mouse models were counterstained with H&E, and high-magnification images of the yellow-boxed area are shown. Scale bars: 250 μm. **(F)** Schematic overview of the experimental design (left panel). Seven-week-old male NOD/SCID mice were intravenously injected with HCT116 cells (1×10^6) through the tail vein. Mice were sacrificed at 3 weeks, and lung segments were fixed by formalin. Lung sections from the tail vein-injected mouse models were counterstained with H&E, and high-magnification images of the yellow-boxed area are shown (right panel). Scale bars: 250 μm.

Figure S2

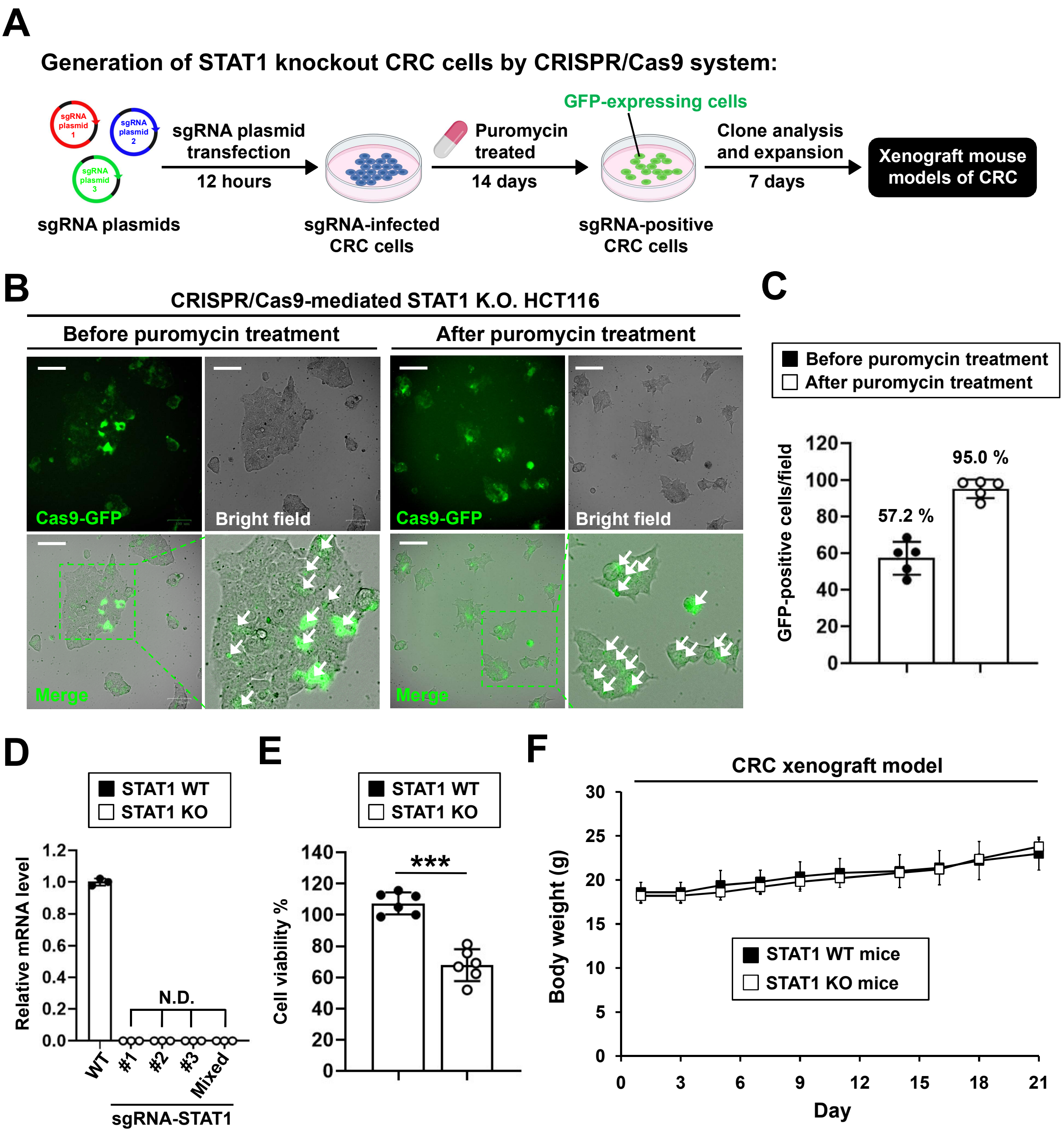

Figure S2. Generation of STAT1 knockout colorectal cancer cells using CRISPR/Cas9 system. (A) Schematic overview of the CRISPR/Cas9 system in CRC cells. CRISPR plasmids expressing Cas9 and STAT1 sgRNAs were transfected into HCT116 cells for 12 hours. Cells were treated with puromycin (0.5 μg/ml) for 14 days. Single clones were picked according to

GFP expression and expanded to generate monoclonal cell colonies. **(B)** Successful transfection of CRISPR/Cas9 KO plasmids could be visually confirmed by detection of the GFP. The white arrowhead indicates the GFP-positive HCT116 cells. Scale bar: 100 μm. **(C)** GFP-positive HCT116 cells were calculated. **(D)** The STAT1 mRNA levels in HCT116 cells treated with STAT1 sgRNAs versus control were quantified by qPCR analysis. The mRNA expression was normalized to GAPDH. N.D., not detected for 40 cycles by qPCR. **(E)** Role of STAT1 on cell viability in HCT116 cells. STAT1 WT cells and STAT1 KO cells were incubated, and cell viability was measured at 48 hours by the MTT assay. ***$p < 0.001$. **(F)** Changes of the body weights in HCT116 xenograft mouse models are shown.

Figure S3

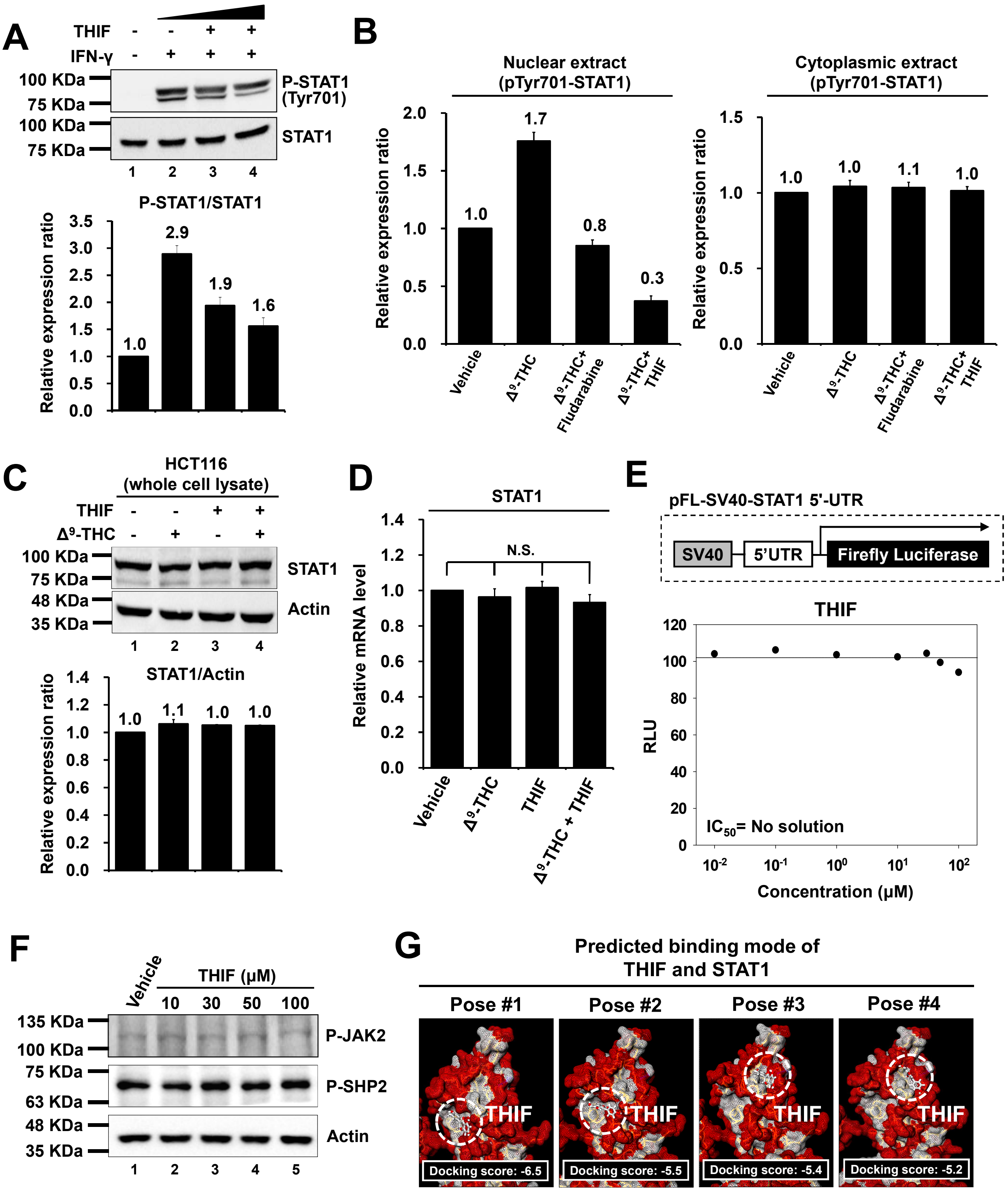
A
THIF - - + +
IFN-γ - + + +
100 KDa
75 KDa
P-STAT1 (Tyr701)
100 KDa
75 KDa
STAT1
1 2 3 4
P-STAT1/STAT1
Relative expression ratio
1.0 2.9 1.9 1.6
B
Nuclear extract (pTyr701-STAT1)
Relative expression ratio
1.0 1.7 0.8 0.3
Vehicle
Δ9-THC
Δ9-THC+ Fludarabine
Δ9-THC+ THIF
Cytoplasmic extract (pTyr701-STAT1)
1.0 1.0 1.1 1.0
C
HCT116 (whole cell lysate)
THIF - - + +
Δ9-THC - + - +
100 KDa
75 KDa
STAT1
48 KDa
35 KDa
Actin
1 2 3 4
STAT1/Actin
Relative expression ratio
1.0 1.1 1.0 1.0
D
STAT1
N.S.
Relative mRNA level
Vehicle
Δ9-THC
THIF
Δ9-THC + THIF
E
pFL-SV40-STAT1 5'-UTR
SV40
5'UTR
Firefly Luciferase
THIF
RLU
IC50= No solution
Concentration (μM)
F
Vehicle
THIF (μM)
10 30 50 100
135 KDa
100 KDa
P-JAK2
75 KDa
63 KDa
P-SHP2
48 KDa
35 KDa
Actin
1 2 3 4 5
G
Predicted binding mode of THIF and STAT1
Pose #1
Pose #2
Pose #3
Pose #4
THIF
Docking score: -6.5
Docking score: -5.5
Docking score: -5.4
Docking score: -5.2

Figure S3 (continue)

H

Receptor−ligand interaction diagrams of THIF and STAT1

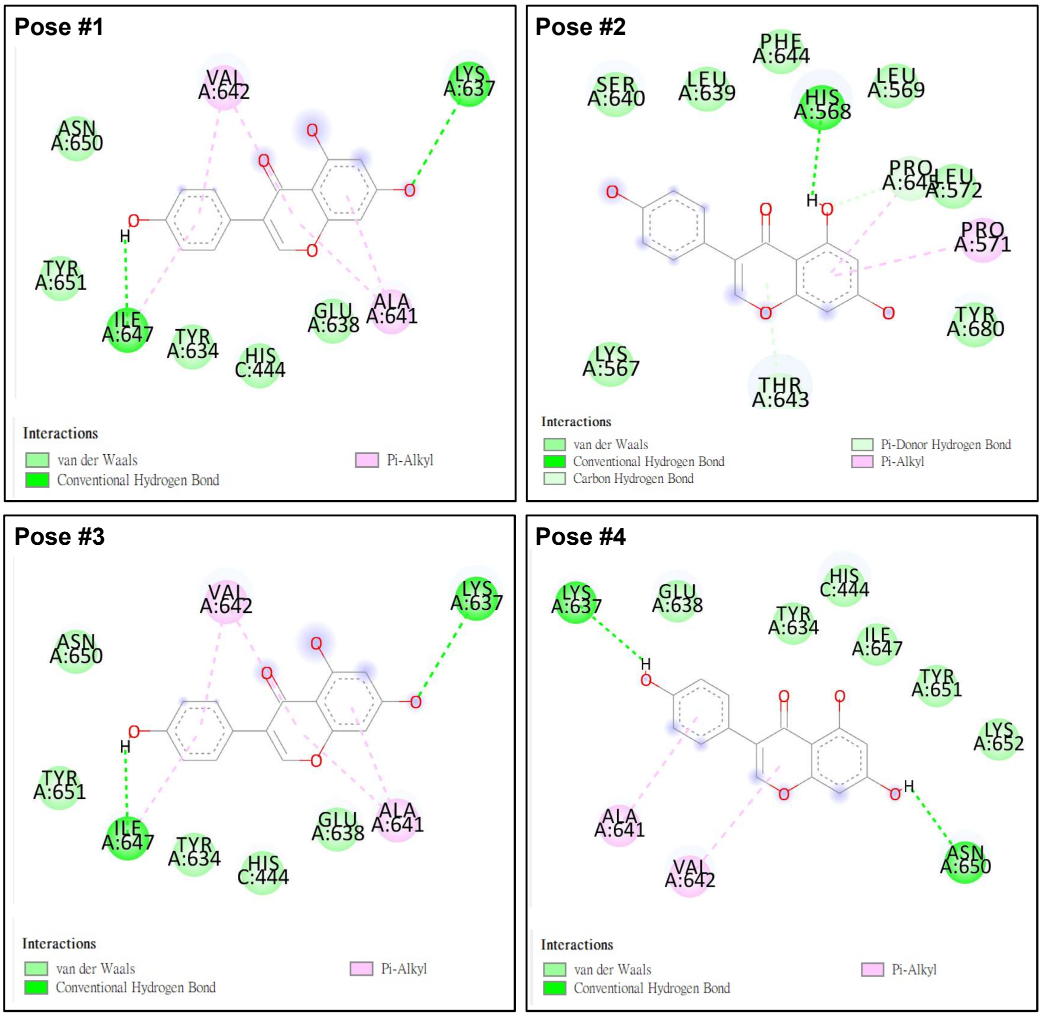

I

Interactions between THIF and amino acid residues of STAT1

	Pose #1	Pose #2	Pose #3	Pose #4
HIS444	V		V	V
LYS567		V		
HIS568		V		
LEU569		V		
PRO571		V		
LEU572		V		
TYR634	V		V	V
LYS637	V		V	V
GLU638	V		V	V
LEU639		V		
SER640		V		
ALA641	V		V	V
VAL642	V		V	V
THR643		V		
PHE644		V		
PRO645		V		
ILE647	V		V	V
ASN650	V		V	V
TYR651	V		V	V
LYS652				V
TYR680		V		

STAT1 SH2 Domain (TYR634–TYR680)

Figure S3. THIF can directly bind to the STAT1 protein. (A) Effect of THIF on STAT1 phosphorylation in CRC cells. HCT116 cells were treated with STAT1 activator (500 ng/ml IFN-γ) or STAT1 activator plus THIF (10 μM or 50 μM) for 12 hours. Total protein extract was prepared and Western blot analysis was performed using indicated antibodies (upper panel). Western blot images were quantified by ImageJ software (lower panel). **(B)** Effect of THIF on STAT1 phosphorylation in CRC cells. HCT116 cells were treated with 5 μM Δ^9-THC, Δ^9-THC plus 10 μM THIF or Δ^9-THC plus 10 μM fludarabine for 12 hours. Protein extracts from the nuclear and cytoplasmic fractions were prepared. Western blot analysis was performed using the indicated antibodies. Western blot images were quantitatively analyzed with ImageJ software. **(C and D)** HCT116 cells were treated with 5 μM Δ^9-THC, 50 μM THIF or their combination for 48 hours. **(C)** Protein extract was prepared and Western blot analysis was performed using indicated antibodies (upper panel). Western blot images were quantified by ImageJ software (lower panel). **(D)** qPCR analysis was performed. The mRNA expression was normalized to GAPDH. N.S., no significance. **(E)** Dose-response effect of THIF on STAT1 activity. The luciferase activity of STAT1 was measured by the STAT1 reporter assay in 293T cells. Cells were treated with increasing concentrations of THIF for 12 hours. The 50% of inhibition concentration (IC_{50}) of luciferase activity is calculated by SigmaPlot software. **(F)** HCT116 cells were treated with indicated concentrations of THIF for 48 hours. Protein extract was prepared and Western blot analysis was performed using indicated antibodies. **(G-H)** Conformations of THIF docked in binding sites on the STAT1 protein. **(G)** Docking models of THIF in the STAT1 crystal structure (PDB: 1YVL). The predicted binding modes of THIF and STAT1 are shown. The white circle indicates the binding site of THIF. **(H)** The binding interactions between THIF and amino acid residues in STAT1 protein are shown. **(I)** The overlapping binding sites of four predicted binding modes of THIF with the STAT1 SH2 domain are highlighted in blue.

Figure S4

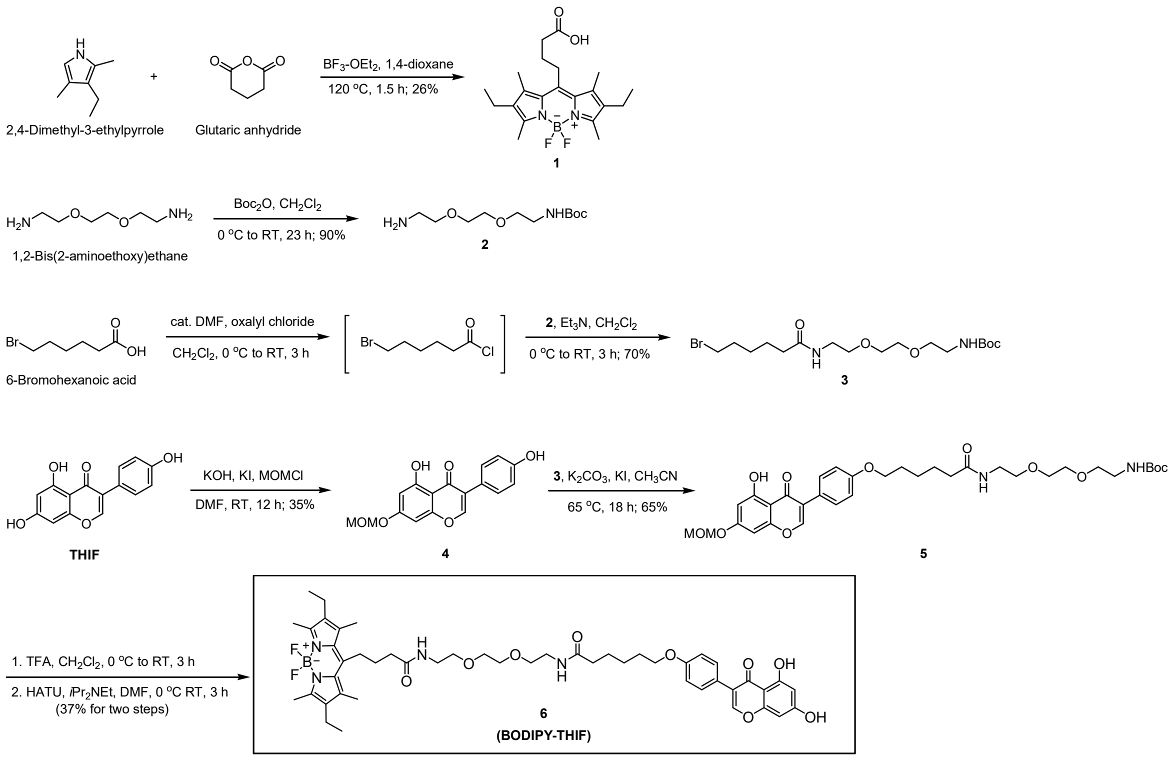

Figure S4. A synthesis plan of fluorescently labeled STAT1 probe inhibitor. Reactions are magnetically stirred and monitored by thin-layer chromatography on silica gel. Flash chromatography is performed on silica gel of 60-200 μm particle size. Yields are reported for spectroscopically pure compounds. Melting points are recorded on a Fargo MP-2D melting point apparatus. 1H, 13C, and 31P NMR spectra are recorded on Bruker AV 600 (600 MHz), Bruker AV 500 (500 MHz) and Bruker AVIII 17 400 (400 MHz) spectrometers. Chemical shifts are given in δ values relative to tetramethylsilane (δH = 0); coupling constants J are given in Hz. Internal standards are CDCl3 (δH = 7.24), CD3OD (δH = 3.31) or DMSO-d6 (δH = 2.49) for 1H NMR spectra and CDCl3 (δC = 77.0, central line of triplet), CD3OD (δC = 49.0, central line of septet) or DMSO-d6 (δC = 39.5, central line of septet) for 13C NMR spectra. The splitting patterns are reported as s (singlet), d (doublet), t (triplet), q (quartet), m (multiplet), br (broad), and dd (double of doublets). High-resolution electrospray ionization and fast atom bombardment mass spectra were recorded on a JMS-T100LP AccuTOF LC-plus 4G mass spectrometer.

Figure S5

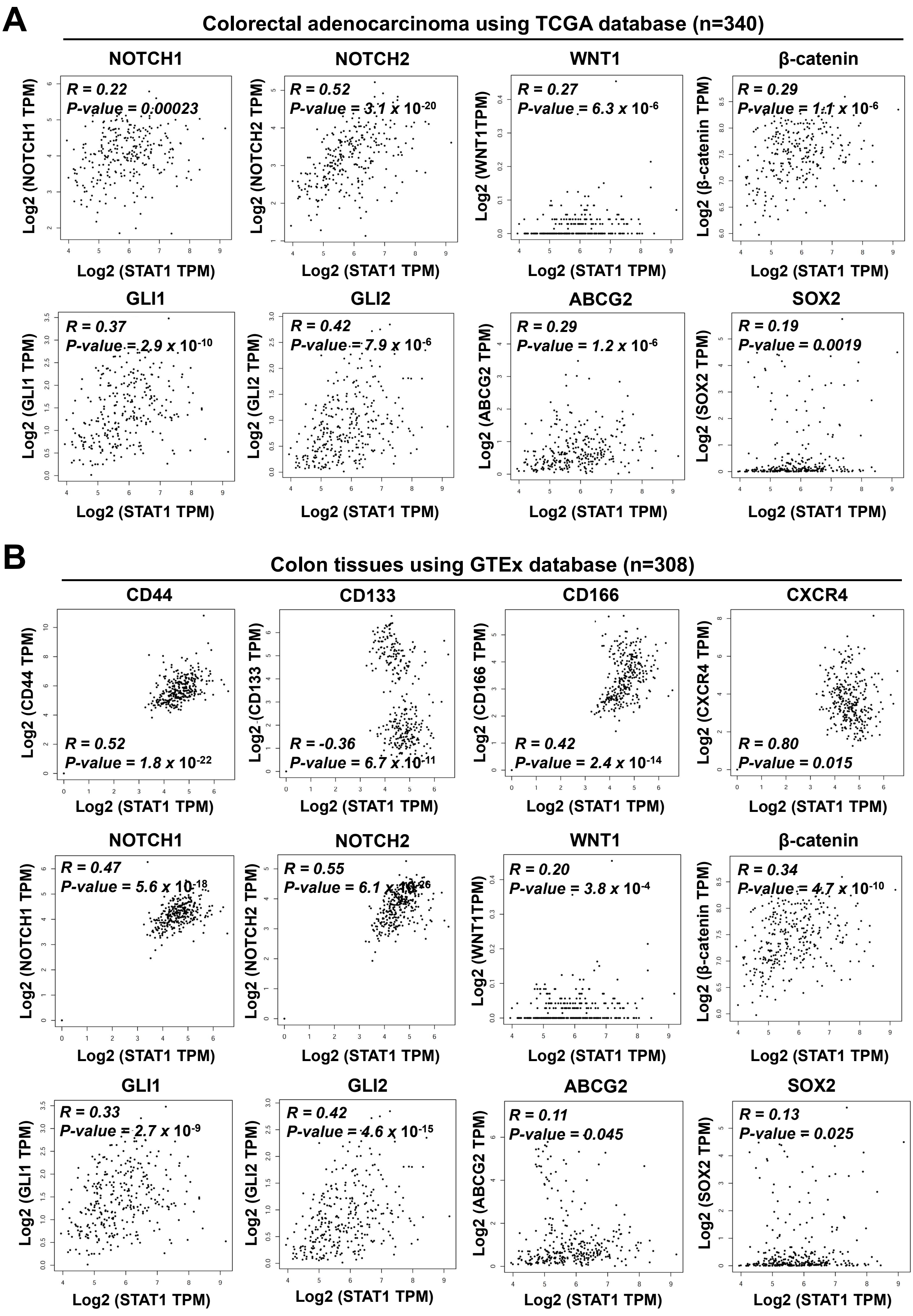
A
Colorectal adenocarcinoma using TCGA database (n=340)
NOTCH1
R = 0.22
P-value = 0.00023
NOTCH2
R = 0.52
P-value = 3.1 x 10^{-20}
WNT1
R = 0.27
P-value = 6.3 x 10^{-6}
β-catenin
R = 0.29
P-value = 1.1 x 10^{-6}
GLI1
R = 0.37
P-value = 2.9 x 10^{-10}
GLI2
R = 0.42
P-value = 7.9 x 10^{-6}
ABCG2
R = 0.29
P-value = 1.2 x 10^{-6}
SOX2
R = 0.19
P-value = 0.0019
Log2 (NOTCH1 TPM)
Log2 (NOTCH2 TPM)
Log2 (WNT1TPM)
Log2 (β-catenin TPM)
Log2 (GLI1 TPM)
Log2 (GLI2 TPM)
Log2 (ABCG2 TPM)
Log2 (SOX2 TPM)
Log2 (STAT1 TPM)
B
Colon tissues using GTEx database (n=308)
CD44
R = 0.52
P-value = 1.8 x 10^{-22}
CD133
R = -0.36
P-value = 6.7 x 10^{-11}
CD166
R = 0.42
P-value = 2.4 x 10^{-14}
CXCR4
R = 0.80
P-value = 0.015
NOTCH1
R = 0.47
P-value = 5.6 x 10^{-18}
NOTCH2
R = 0.55
P-value = 6.1 x 10^{-26}
WNT1
R = 0.20
P-value = 3.8 x 10^{-4}
β-catenin
R = 0.34
P-value = 4.7 x 10^{-10}
GLI1
R = 0.33
P-value = 2.7 x 10^{-9}
GLI2
R = 0.42
P-value = 4.6 x 10^{-15}
ABCG2
R = 0.11
P-value = 0.045
SOX2
R = 0.13
P-value = 0.025
Log2 (CD44 TPM)
Log2 (CD133 TPM)
Log2 (CD166 TPM)
Log2 (CXCR4 TPM)
Log2 (NOTCH1 TPM)
Log2 (NOTCH2 TPM)
Log2 (WNT1TPM)
Log2 (β-catenin TPM)
Log2 (GLI1 TPM)
Log2 (GLI2 TPM)
Log2 (ABCG2 TPM)
Log2 (SOX2 TPM)
Log2 (STAT1 TPM)

Figure S5 (continue)

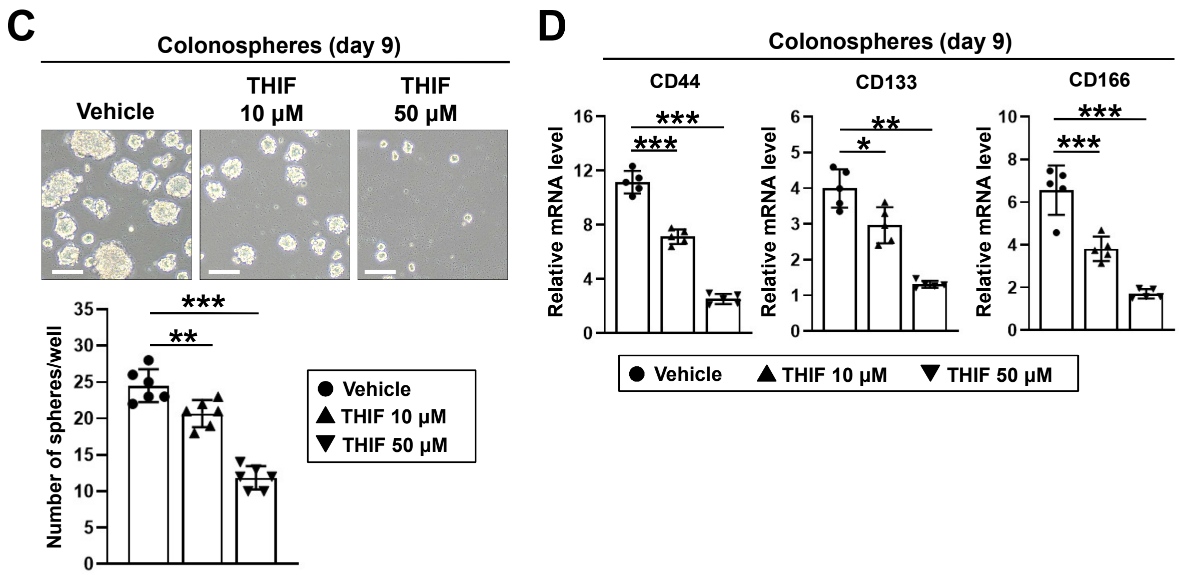

Figure S5. Effect of THIF on cancer stemness. (A) Correlations between STAT1 and common cancer stem cell (CSC) markers are shown in patients with colorectal adenocarcinoma using TCGA database (https://gdc.cancer.gov/). **(B)** Correlations between STAT1 and CSC markers are shown in human colon tissues using the GTEx database (https://www.gtexportal.org/home/). **(C and D)** HCT116 colonospheres were dissociated into single cells and plated at a density of 5 × 10^4 cells/well in a 6-well Ultra-Low plate. Cells were treated with 10 or 50 μM THIF for 48 hours. **(C)** Cell morphology was monitored by microscopy (upper panel). Scale bars: 100 μm. The number of colospheres was calculated (lower panel). ***p*< 0.01; ****p*< 0.001. **(D)** The mRNA expression of stemness genes was measured by qPCR. The mRNA expression was normalized to GAPDH. **p*< 0.05; ***p*< 0.01; ****p*< 0.001.

Figure S6

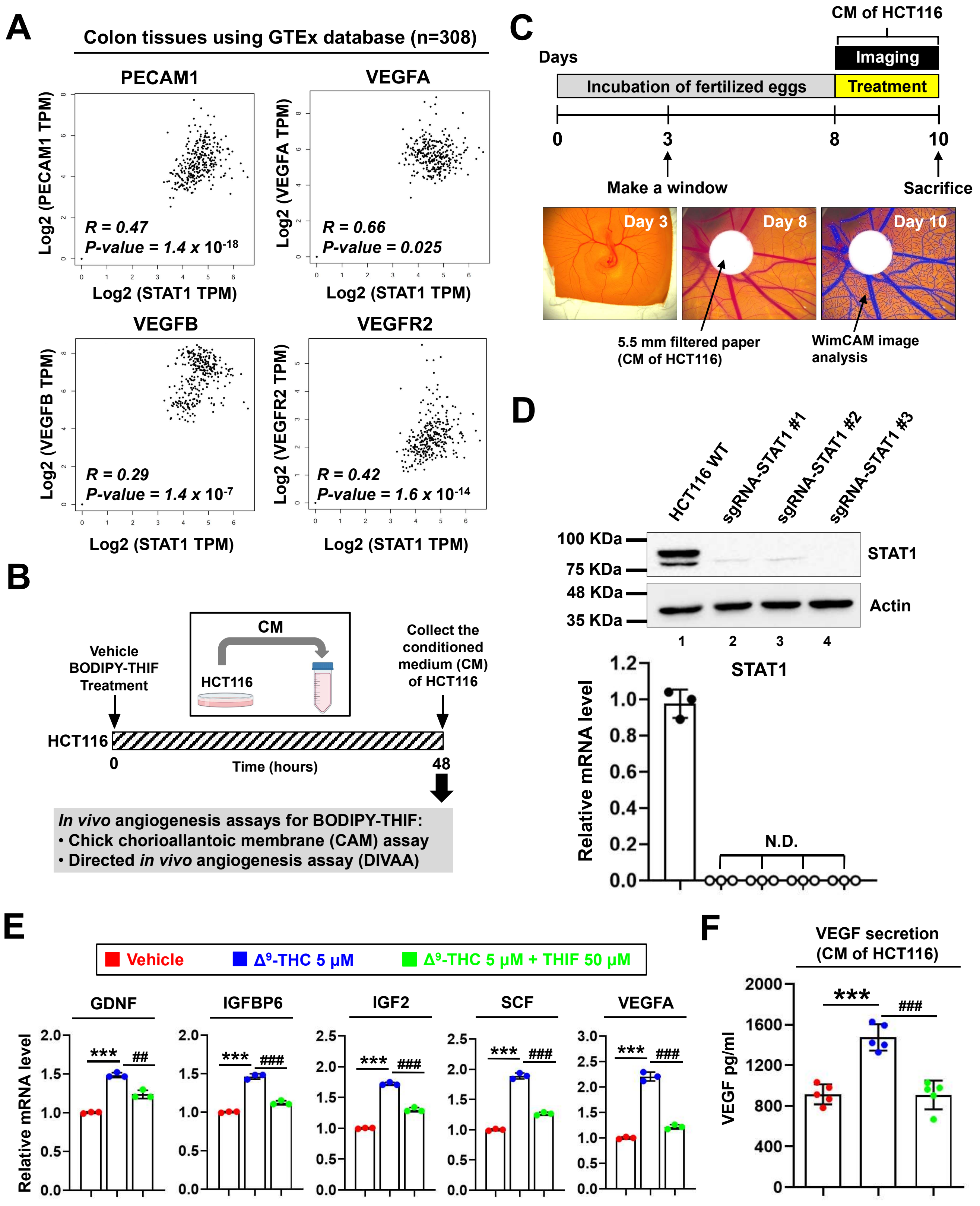
A
Colon tissues using GTEx database (n=308)
PECAM1
Log2 (PECAM1 TPM)
R = 0.47
P-value = 1.4 x 10-18
Log2 (STAT1 TPM)
VEGFA
Log2 (VEGFA TPM)
R = 0.66
P-value = 0.025
Log2 (STAT1 TPM)
VEGFB
Log2 (VEGFB TPM)
R = 0.29
P-value = 1.4 x 10-7
Log2 (STAT1 TPM)
VEGFR2
Log2 (VEGFR2 TPM)
R = 0.42
P-value = 1.6 x 10-14
Log2 (STAT1 TPM)
B
Vehicle
BODIPY-THIF
Treatment
CM
HCT116
Collect the conditioned medium (CM) of HCT116
HCT116
0
Time (hours)
48
In vivo angiogenesis assays for BODIPY-THIF:
• Chick chorioallantoic membrane (CAM) assay
• Directed in vivo angiogenesis assay (DIVAA)
C
CM of HCT116
Days
Imaging
Incubation of fertilized eggs
Treatment
0
3
8
10
Make a window
Sacrifice
Day 3
Day 8
Day 10
5.5 mm filtered paper (CM of HCT116)
WimCAM image analysis
D
HCT116 WT
sgRNA-STAT1 #1
sgRNA-STAT1 #2
sgRNA-STAT1 #3
100 KDa
75 KDa
48 KDa
35 KDa
STAT1
Actin
1
2
3
4
STAT1
Relative mRNA level
N.D.
E
Vehicle
Δ9-THC 5 μM
Δ9-THC 5 μM + THIF 50 μM
GDNF
IGFBP6
IGF2
SCF
VEGFA
Relative mRNA level
***
##
###
F
VEGF secretion (CM of HCT116)
VEGF pg/ml
***
###

Figure S6. Effect of THIF on angiogenesis in colorectal cancer *in vitro* and *in vivo*. **(A)** Correlations between STAT1 and angiogenesis-related markers are shown in human colon tissues using the GTEx database. **(B)** Schematic overview of the experimental design for *in vivo* angiogenesis assays. **(C)** HCT116 cells were treated with vehicle (control) or BODIPY-THIF (50 μM or 100 μM) for 48 hours, and the CM was collected for the chick chorioallantoic membrane (CAM) assay. Sterilized filter-paper disks were used as a carrier for CM of HCT116 cells. Images were quantified by WimCAM image analysis software. **(D)** Validation of STAT1 expression by Q-PCR and western blot analysis in STAT1 KO cells. The STAT1 protein levels and mRNA levels in HCT116 cells treated with STAT1 sgRNAs versus control were quantified by Western blot analysis and Q-PCR analysis. Protein extract was prepared and Western blot analysis was performed using indicated antibodies (upper panel). mRNA expression was normalized to GAPDH (lower panel). N.D., not detected for 40 cycles by Q-PCR. Data are representative of three independent experiments and values are expressed in mean ± SD. **(E)** Effect of THIF on Δ^9-THC-induced angiogenesis in CRC cells. HCT116 cells were treated with 5 μM Δ^9-THC or 5 μM Δ^9-THC plus 50 μM THIF for 48 hours. The mRNA expressions of the five angiogenesis-related genes were quantified by qPCR analysis and normalized to GAPDH. $^{***}p < 0.001$ versus vehicle; $^{\#\#}p < 0.01$ versus Δ^9-THC; $^{\#\#\#}p < 0.001$ versus Δ^9-THC. **(F)** THIF reduces the release of VEGF from CRC cells after Δ^9-THC treatment. HCT116 cells were treated with 5 μM Δ^9-THC or 5 μM Δ^9-THC plus 50 μM THIF for 48 hours, and VEGF concentration in conditioned medium (CM) was measured by ELISA. $^{***}p < 0.001$ versus vehicle; $^{\#\#\#}p < 0.001$ versus Δ^9-THC.

Figure S7

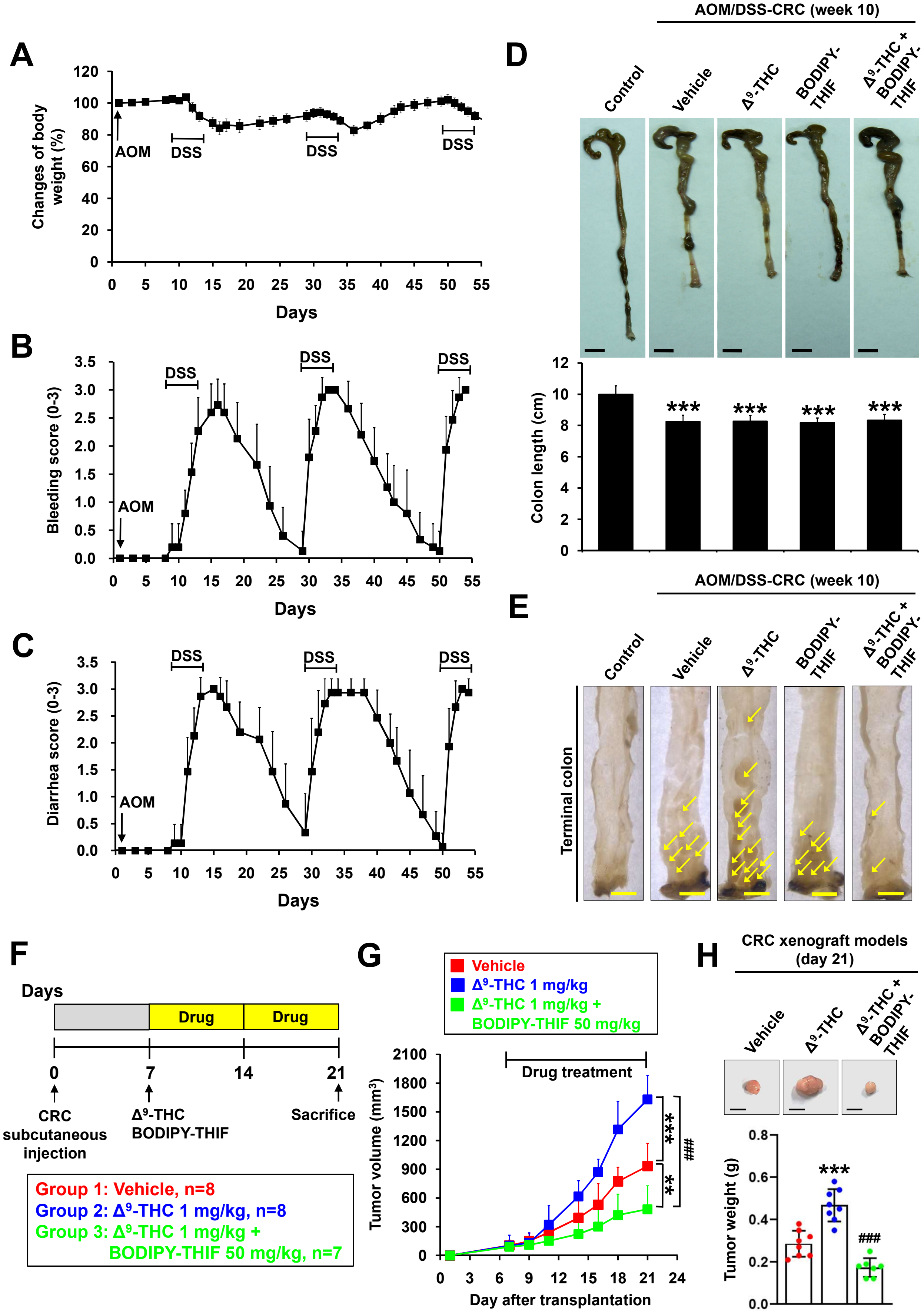
A
Changes of body weight (%)
AOM
DSS
DSS
DSS
Days
B
Bleeding score (0-3)
AOM
DSS
DSS
DSS
Days
C
Diarrhea score (0-3)
AOM
DSS
DSS
DSS
Days
AOM/DSS-CRC (week 10)
D
Control
Vehicle
Δ⁹-THC
BODIPY-THIF
Δ⁹-THC + BODIPY-THIF
Colon length (cm)
***
AOM/DSS-CRC (week 10)
E
Terminal colon
F
Days
Drug
Drug
CRC subcutaneous injection
Δ⁹-THC BODIPY-THIF
Sacrifice
Group 1: Vehicle, n=8
Group 2: Δ⁹-THC 1 mg/kg, n=8
Group 3: Δ⁹-THC 1 mg/kg + BODIPY-THIF 50 mg/kg, n=7
G
Vehicle
Δ⁹-THC 1 mg/kg
Δ⁹-THC 1 mg/kg + BODIPY-THIF 50 mg/kg
Drug treatment
Tumor volume (mm³)
Day after transplantation
***
**
###
H
CRC xenograft models (day 21)
Vehicle
Δ⁹-THC
Δ⁹-THC + BODIPY-THIF
Tumor weight (g)
***
###

Figure S7. Effects of BODIPY-THIF on tumor growth in AOM/DSS-induced colorectal cancer mouse models. CRC was induced by intraperitoneal injection of AOM (12.5 mg/kg) in conjunction with the DSS stimulus. Mice were maintained with a regular diet and drinking water for 7 days and then subjecting to 3 cycles of DSS treatment, with each cycle consisting of the administration of 3.5% DSS for 5 days followed by a 14-day recovery period with regular water. **(A-C)** Colitis symptom in AOM/DSS mouse models during the CRC induction and recovery phases. **(A)** Changes in body weights are shown. **(B)** Clinical bleeding scores are shown. **(C)** Clinical diarrhea scores are shown. **(D)** Representative whole colons (upper panel) and colon length (lower panel) are shown. Scale bar, 2 cm. ***p< 0.001 versus healthy control. **(E)** Representative whole colons are depicted, and the yellow arrowhead indicates macroscopic polyps. Scale bars: 5 mm. **(F-H)** Effect of BODIPY-THIF on colorectal tumor growth in xenograft mouse models. HCT116 cells (1×10^7 cells) were subcutaneously implanted into male nude mice. When the tumor volume reached 100 mm^3, mice were treated with vehicle (control), Δ^9-THC (1 mg/kg, intraperitoneally), or Δ^9-THC (1 mg/kg, intraperitoneally) plus BODIPY-THIF (50 mg/kg, orally) every two days for two weeks. The tumor volume was measured every two days after drug treatment. **(F)** Schematic overview of the experimental design in the mouse model. **(G)** The tumor volume was calculated as follows: $V = 0.5 \times$ (the length of length) $\times$ (the length of width)^2. **p< 0.01 versus vehicle; ***p< 0.001 versus vehicle; $^{\#\#\#}p < 0.001$ versus Δ^9-THC. **(H)** Gross pictures of xenograft tumors are shown (upper panel). Scale bar: 1 cm. At the end of the experiment, the excised tumors were weighed (lower panel). ***p< 0.001 versus vehicle; $^{\#\#\#}p < 0.001$ versus Δ^9-THC.

Figure S8

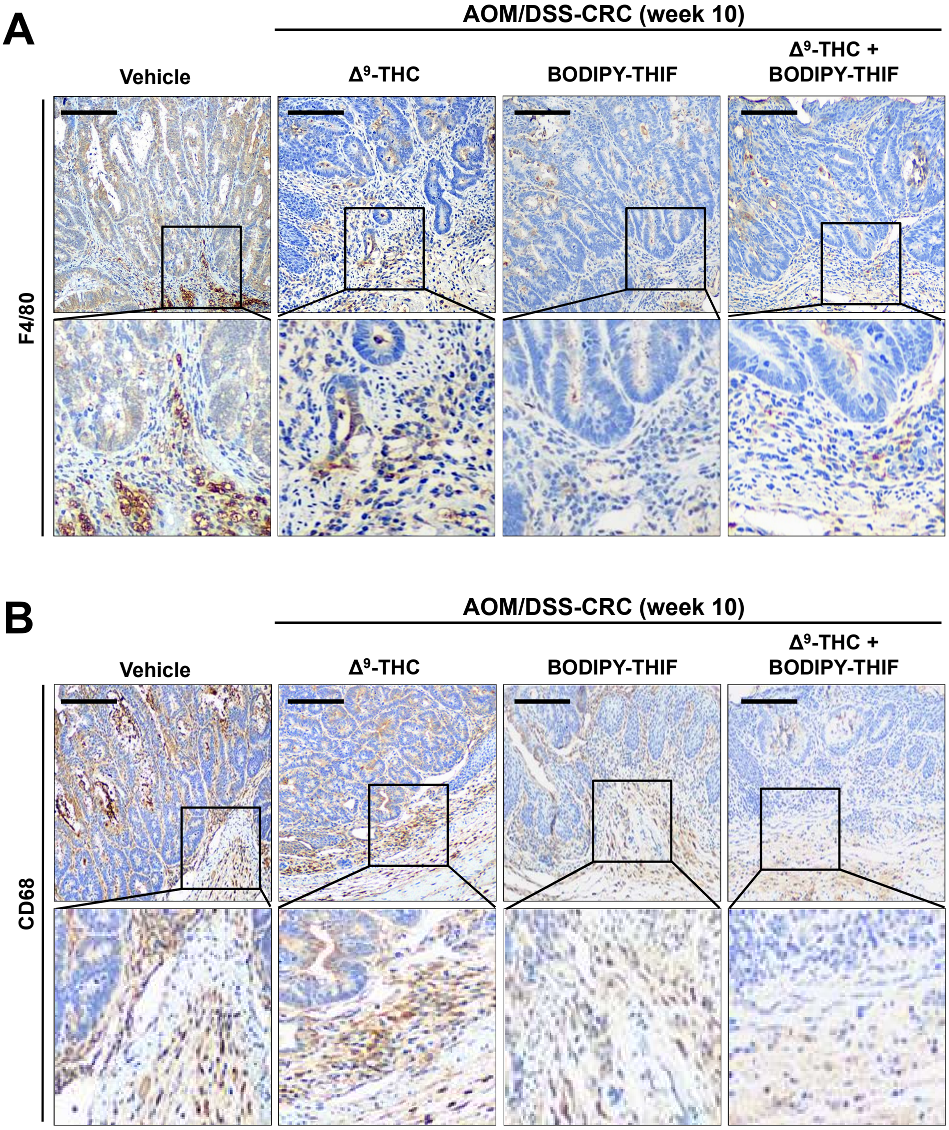

Figure 8. Effects of STAT1 on immune response in colorectal cancer. AOM/DSS-treated mice were randomly divided into four groups. Mice were treated with vehicle (control), Δ^9-THC (1 mg/kg, intraperitoneally), BODIPY-THIF (50 mg/kg, orally), or Δ^9-THC (1 mg/kg, intraperitoneally) plus BODIPY-THIF (50 mg/kg, orally) five days a week for two weeks after AOM/DSS treatment. Colon sections were immunostained with anti-F4/80 **(A)** and anti-CD68 **(B)** antibodies, and one representative experiment of three was presented. High-magnification images of the areas in the black boxes are shown. Scale bar, 250 μm.

Table S1. List of antibodies used in this study.

ANTIBODIES	SUPPLIER NAME	CATALOG NUMBER
Anti-STAT1antibody	Cell Signaling	#9172
Anti-phospho-STAT1 (Tyr701) antibody	Cell Signaling	#9167
Anti-α-Tubulin antibody	Abcam	ab7291
Anti-lamin B1 antibody	Cell Signaling	#12586
Anti-phospho-SHP-2 (Tyr580) antibody	Abcam	ab75818
Anti-phospho-JAK2 (Tyr1007/1008) antibody	ABclonal	AP0531
Anti-F4/80 antibody	Abcam	ab111101
Anti-CD68 antibody	Bio-Red	MCA1957
Anti-β-actin antibody	GeneTex	GTX109639
Rabbit IgG, polyclonal - Isotype Control	Abcam	ab37415
Rabbit IgG - Normal Control	Santa Cruz	sc-2027

Table S2. Predicted protein targets of THIF from the SwissTargetPrediction Database.

TARGETS [a]	TARGET CLASS
Thromboxane-A synthase	Cytochrome P450
Monoamine oxidase A	Oxidoreductase
Epidermal growth factor receptor erbB1	Kinase
Estrogen receptor alpha	Nuclear receptor
Maltase-glucoamylase	Hydrolase
Serotonin 2a (5-HT2a) receptor	Family A G protein-coupled receptor
Serotonin 2c (5-HT2c) receptor	Family A G protein-coupled receptor
Adenosine A1 receptor	Family A G protein-coupled receptor
Estrogen receptor beta	Nuclear receptor
Adenosine A2a receptor	Family A G protein-coupled receptor

[a] Top ten predicted protein targets of THIF from the Swiss Target Prediction Database (http://www.swisstargetprediction.ch/).
